# Non-invasive measurements of ictal and interictal epileptiform activity using optically pumped magnetometers

**DOI:** 10.1038/s41598-023-31111-y

**Published:** 2023-03-21

**Authors:** Arjan Hillebrand, Niall Holmes, Ndedi Sijsma, George C. O’Neill, Tim M. Tierney, Niels Liberton, Anine H. Stam, Nicole van Klink, Cornelis J. Stam, Richard Bowtell, Matthew J. Brookes, Gareth R. Barnes

**Affiliations:** 1 Department of Clinical Neurophysiology and Magnetoencephalography Center, Amsterdam UMC Location Vrije Universiteit Amsterdam, De Boelelaan 1117, 1081HV Amsterdam, The Netherlands; 2 Brain Imaging, Amsterdam Neuroscience, Amsterdam, The Netherlands; 3 Systems and Network Neurosciences, Amsterdam Neuroscience, Amsterdam, The Netherlands; 4 Sir Peter Mansfield Imaging Centre, School of Physics and Astronomy, University of Nottingham, University Park, Nottingham, NG7 2RD UK; 5 Wellcome Centre for Human Neuroimaging, Department of Imaging Neuroscience, UCL Queen Square Institute of Neurology, University College London, London, WC1N 3AR UK; 6 Department of Medical Technology, 3D Innovation Lab, Amsterdam UMC Location Vrije Universiteit Amsterdam, Amsterdam, The Netherlands; 7 Department of Neurology and Neurosurgery, UMC Utrecht Brain Center, University Medical Center Utrecht, Heidelberglaan 100, 3584 CX Utrecht, The Netherlands; 8 Neurodegeneration, Amsterdam Neuroscience, Amsterdam, The Netherlands

**Keywords:** Epilepsy, Magnetoencephalography

## Abstract

Magneto- and electroencephalography (MEG/EEG) are important techniques for the diagnosis and pre-surgical evaluation of epilepsy. Yet, in current cryogen-based MEG systems the sensors are offset from the scalp, which limits the signal-to-noise ratio (SNR) and thereby the sensitivity to activity from deep structures such as the hippocampus. This effect is amplified in children, for whom adult-sized fixed-helmet systems are typically too big. Moreover, ictal recordings with fixed-helmet systems are problematic because of limited movement tolerance and/or logistical considerations. Optically Pumped Magnetometers (OPMs) can be placed directly on the scalp, thereby improving SNR and enabling recordings during seizures. We aimed to demonstrate the performance of OPMs in a clinical population. Seven patients with challenging cases of epilepsy underwent MEG recordings using a 12-channel OPM-system and a 306-channel cryogen-based whole-head system: three adults with known deep or weak (low SNR) sources of interictal epileptiform discharges (IEDs), along with three children with focal epilepsy and one adult with frequent seizures. The consistency of the recorded IEDs across the two systems was assessed. In one patient the OPMs detected IEDs that were not found with the SQUID-system, and in two patients no IEDs were found with either system. For the other patients the OPM data were remarkably consistent with the data from the cryogenic system, noting that these were recorded in different sessions, with comparable SNRs and IED-yields overall. Importantly, the wearability of OPMs enabled the recording of seizure activity in a patient with hyperkinetic movements during the seizure. The observed ictal onset and semiology were in agreement with previous video- and stereo-EEG recordings. The relatively affordable technology, in combination with reduced running and maintenance costs, means that OPM-based MEG could be used more widely than current MEG systems, and may become an affordable alternative to scalp EEG, with the potential benefits of increased spatial accuracy, reduced sensitivity to volume conduction/field spread, and increased sensitivity to deep sources. Wearable MEG thus provides an unprecedented opportunity for epilepsy, and given its patient-friendliness, we envisage that it will not only be used for presurgical evaluation of epilepsy patients, but also for diagnosis after a first seizure.

## Introduction

Magneto- and Electroencephalography (MEG/EEG) are important techniques for the diagnosis^1^ and pre-surgical evaluation^2,3^ of epilepsy. For patients with focal refractory epilepsy, seizure freedom can be achieved through epilepsy surgery by removing the epileptogenic zone (EZ), which is defined as the area of cortex that is necessary and sufficient for initiating seizures and whose removal (or disconnection) is necessary for complete abolition of seizures^4^. This requires the generation of a hypothesis about the location of the EZ during the pre-surgical workup using measurements from non-invasive techniques such as MEG/EEG, or invasive recordings using intracranial electrodes^5^. Interictal epileptiform discharges (IEDs) and ictal activity as identified in presurgical MEG/EEG help to identify the irritative zone (the area of cortical tissue that generates IEDs) and the seizure onset zone (the area of cortex from which clinical seizures are generated), respectively, both of which may overlap with the EZ^6,7^. The pre-surgical workup, and thereby surgery, needs to be improved though, as seizure freedom is currently achieved in only two-thirds of the patients who undergo surgery^8,9^.

Current techniques have their limitations: invasive EEG has limited spatial coverage, is burdensome to the patient, expensive, has risk of complications, and may still be inconclusive^10^. Clinical scalp-EEG recordings have limited spatial resolution, which can be mitigated to some extent with high-density recordings in combination with advanced head- and source-modelling^11,12^. Although MEG generally has good spatial resolution^13,14^, its sensitivity to activity from deep structures could improve when recordings with higher signal-to-noise ratios (SNRs) are available^15^. For data-dependent inversion schemes (such as beamforming) SNR also impacts spatial resolution^16^. In current cryogen-based SQUID (Superconducting Quantum Interference Device) systems the SNR and spatial resolution is ultimately constrained by the distance between the scalp and sensors that is required for thermal insulation. As these fixed-helmet systems are typically designed for adults (but see e.g. Ref.^17^), SNR and spatial resolution are further decreased when recording in children. In addition, ictal recordings with SQUID-based helmet systems are often problematic due to movement artefacts, and because long-term observations are not feasible. The ability to record seizure activity is of clinical importance, as this provides the most reliable information with regards to the location of the EZ.

Newly developed, cryogen-free, MEG sensors—Optically Pumped Magnetometers (OPMs)—provide an unprecedented opportunity for epilepsy, since they enable non-invasive, wearable recordings with whole-head sensitivity for ictal and interictal activity. OPMs are small and lightweight, yet have a sensitivity that is comparable to that of SQUIDs^18^. Importantly, they can be placed directly on the scalp^19–21^, which could allow recordings to be made during the ictal period, and also open up the possibility for long-term observations^22^.

OPM sensors are passive magnetic field sensors: the transmission of laser light through a gas cell containing a vapour of spin-polarised rubidium atoms reduces in the presence of an external magnetic field, thus providing a highly sensitive measure of the local magnetic field^23^. The technique of using alkali vapour cell OPMs to measure magnetic fields is over five decades old^24^, yet in the last decade, since the innovation of high performance semiconductor lasers and miniaturisation of optics, OPMs have been miniaturised and commercialised to a footprint and performance suitable for MEG. The viability of the OPM technology in healthy human subjects has recently been demonstrated^25^, and this breakthrough work has been followed-up by an increasing body of work with OPMs^23,26^, including, for example, their use in children^27^, assessment of sensory and motor modalities^28–31^, language lateralization and localisation^32^, speech processing^33^, and the estimation of functional interactions between brain regions^34^. The potential advantages of OPMs in a clinical setting have also been recognized, with several groups demonstrating their utility in epilepsy. Alem and colleagues used OPMs to record IEDs in a rat model of epilepsy^22^, with conformation from intracranial electrical recordings. Feasibility in humans has been demonstrated with a single adult patient^35^. However, ictal recordings and a direct comparison with SQUID-MEG recordings were not performed. More recently, Feys and colleagues reported on the relative merits of OPMs in childhood epilepsy, showing that compared to SQUIDs the IEDs recorded with OPMs had higher SNR for 4 out of the 5 children studied, and higher amplitude for all^36^, although it has yet to be demonstrated that these improvements are also clinically relevant^37^.

The theoretical advantages of OPMs over SQUID-based MEG, which include higher SNRs^36,38,39^ and more accurate source reconstructions^38,40^ have been demonstrated in modelling^38,39^ and (for SNR) experimental^36,40^ studies. To demonstrate the performance of OPMs in a clinical setting we studied six patients with challenging cases of focal, drug-resistant epilepsy, with previously characterised sources of IEDs: three adults with deep or weak (low SNR) sources, and three children with focal epilepsy, in order to demonstrate that the performance with on-scalp sensors is comparable with that of a cryogenic system. A seventh, adult patient with frequent seizures was also included in order to demonstrate that a wearable MEG system enables seizure-recordings.

## Methods

### Patients

We included seven patients with focal, drug-resistant epilepsy who had already undergone a successful clinical SQUID-based MEG at the Amsterdam UMC, location Vrije Universiteit Amsterdam, as part of their clinical workup for epilepsy surgery. The clinical SQUID-based MEG was deemed successful if the patient did not have claustrophobic or anxiety experiences, was cooperative and not restless, and did not cause many artefacts due to e.g. orthodontic material, and if IEDs could be identified. Three children 10–12 years of age were included. The four adult patients had also undergone invasive EEG recordings (stereo-EEG; sEEG), that were used to confirm the irritative zone as identified with MEG. One patient was selected because he had daily seizures. sEEG, in combination with seizure semiology, was used to confirm the seizure onset zone for this patient. None of these patients had undergone surgery for their epilepsy, because the hypothesised EZ was either bilateral in the mesial temporal lobes, multifocal, or near somatosensory areas, or for socioeconomic reasons. Patients used their regular anti-seizure medication on the day of the recordings, and were sleep deprived in order to increase the incidence of IEDs^41^ (see Table S1 for further details, also including patient characteristics and location of IEDs in MEG and EEG, ictal EEG onset, interictal and ictal sEEG findings, PET and CT abnormalities, MRI findings, and semiology). Written informed consent was obtained from patients and/or their caretakers at inclusion, and the study was performed in accordance with the Declaration of Helsinki and approved by the VUmc Medical Ethics Committee.

### OPM setup, recordings, and analyses

#### OPM sensors

Six, commercially available Gen2.0 OPMs (QuSpin Inc, Louisville, CO, USA; Zero field magnetometer, 2nd Generation, dual-axis measurement), with sensitivities of 7–13 fT/√Hz, a dynamic range of ± 5 nT, and a bandwidth of 0 to ~ 130 Hz, were used^18^. We recorded simultaneously along both the radial and a tangential axis to increase the number of measurements and to increase the separability of neuronal and noise signals^42–46^, at a cost of a slight reduction in sensitivity^18^. The sensors operate in the spin exchange relaxation-free regime, and the (near) zero-field environment is achieved through ‘on-sensor’ electromagnetic coils wrapped around the vapour cell, that can compensate for remnant fields in the magnetically shielded room (MSR) of up to 50 nT. Before a recording was started, these coils were activated and optimised for a fixed sensor position and orientation, using QuSpin’s QZFM UI acquisition software (version 6.5.10; using [X, Y, Z]-field zeroing and analog output gain of 0.33). Data were recorded with a sampling frequency of 600 Hz using a National Instruments 16-bit NI-9205 ADC interfaced with a LabVIEW (National Instruments (NI) Corporation, Austin, TX) programme developed at the University of Nottingham. The same software was also used to control coil-drivers (QuSpin Inc), using a 16-bit NI-9264 DAC module, for dynamic noise compensation (see below).

#### Noise compensation

**Figure 1:**
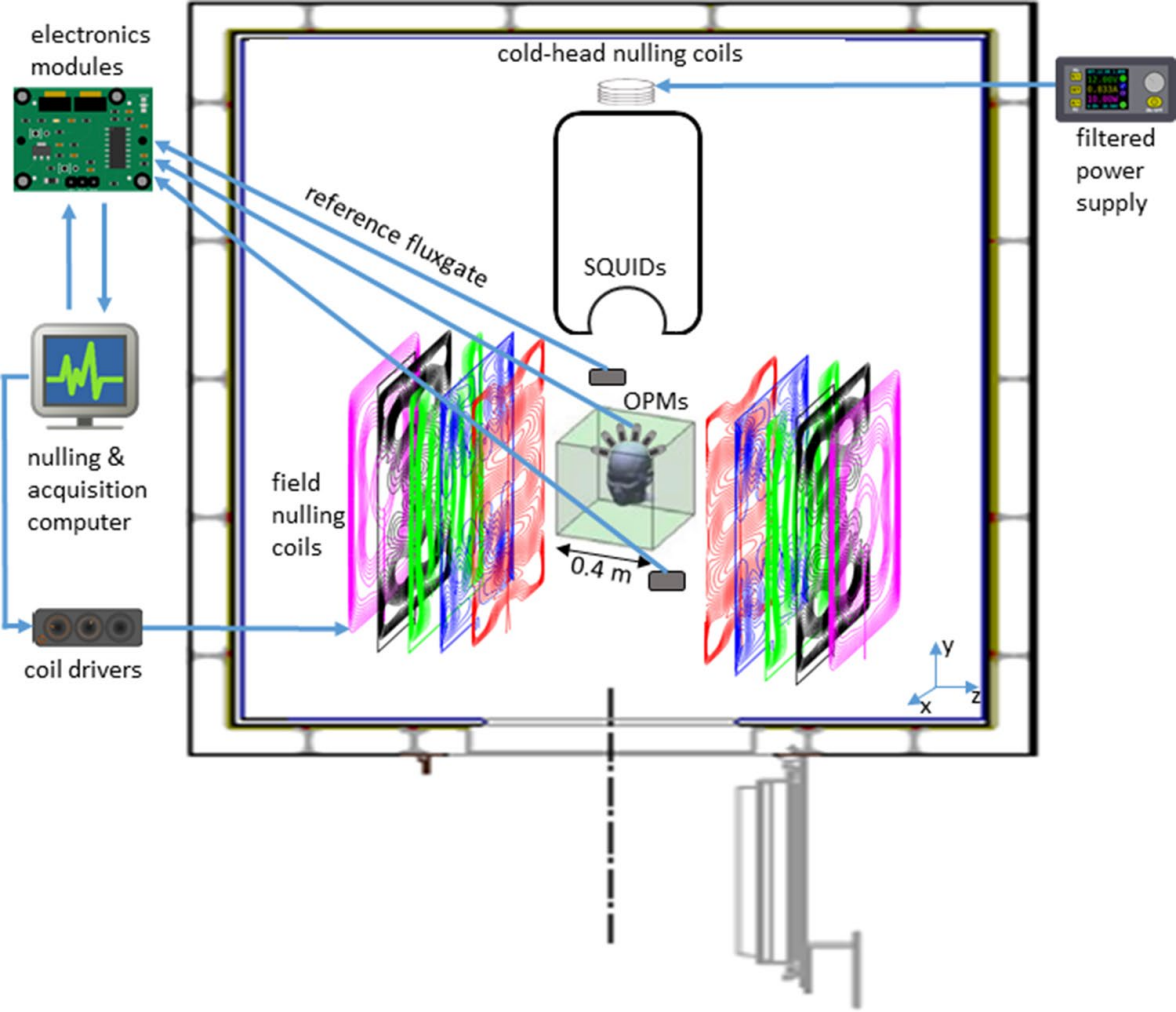
System setup. The whole OPM-system was placed in a magnetically shielded room, together with the SQUID-based system. The cold-head, which is part of the helium liquefier of the SQUID-system, caused static fields with a magnitude of ~ 300 nT. Compensation coils around the cold-head reduced these fields to ~ 30 nT. Field-nulling coils were wound on five large planes placed either side of the participant, different coloured wirepaths show coils designed to produce different field components (shown deliberately offset here; see also Fig. S1). Two fluxgates, placed near the location of where the patient’s head will be during the recordings, were used to record the remnant (static) background fields, and the user manually adjusted the current through the field-nulling coils in order to bring the remnant field level down to ~ 1 nT. During the patient-recordings, the low-pass (< 3 Hz) filtered signals from the OPMs themselves were used to dynamically compensate for temporal variations in the remnant fields, so that the field experienced by the OPMs in a typical recording remained below 0.4 nT.

All MEG recordings were performed inside the MSR (Vacuumschmelze GmbH, Hanau, Germany) at the Department of Clinical Neurophysiology at the VUmc. The MSR houses both the OPM-system and a 306-channel cryogenic system (Triux Neo; MEGIN OY, Espoo, Finland). The cold-head, which is part of the internal helium liquefier of the cryogenic system, causes static fields with a magnitude of ~ 300 nT, which is outside the OPMs operational range of 50 nT. We therefore installed a set of coils around the cold-head (see Supplemental Material) to minimise the field and gradients in the direction of the long-wall of the MSR (Fig. 1). The magnitude of the remnant fields in the MSR was reduced to ~ 30 nT, using a maximum current of 4 A (to avoid overheating) from a low-noise power supply (HMP2020; Rohde & Schwarz GmbH & Co. KG, Munich), fed through an RC lowpass filter unit (− 3 dB at 0.2 Hz) to reduce current noise. A set of 5 bi-planar coils^47^ was then used to bring the remnant fields within the dynamic range of the OPMs (Fig. S1). This coil set generates the three uniform field components and five (linear) magnetic field gradient components to produce a magnetic field which is equal and opposite to that experienced by the OPM array. To compensate the field, two three-axis fluxgates (Bartington Instruments Ltd, Witney, UK; MAG-13MSQ100) were used as reference sensors, and placed at two diagonally opposite corners of the virtual nulling-volume (Fig. 1).

**Figure 2:**
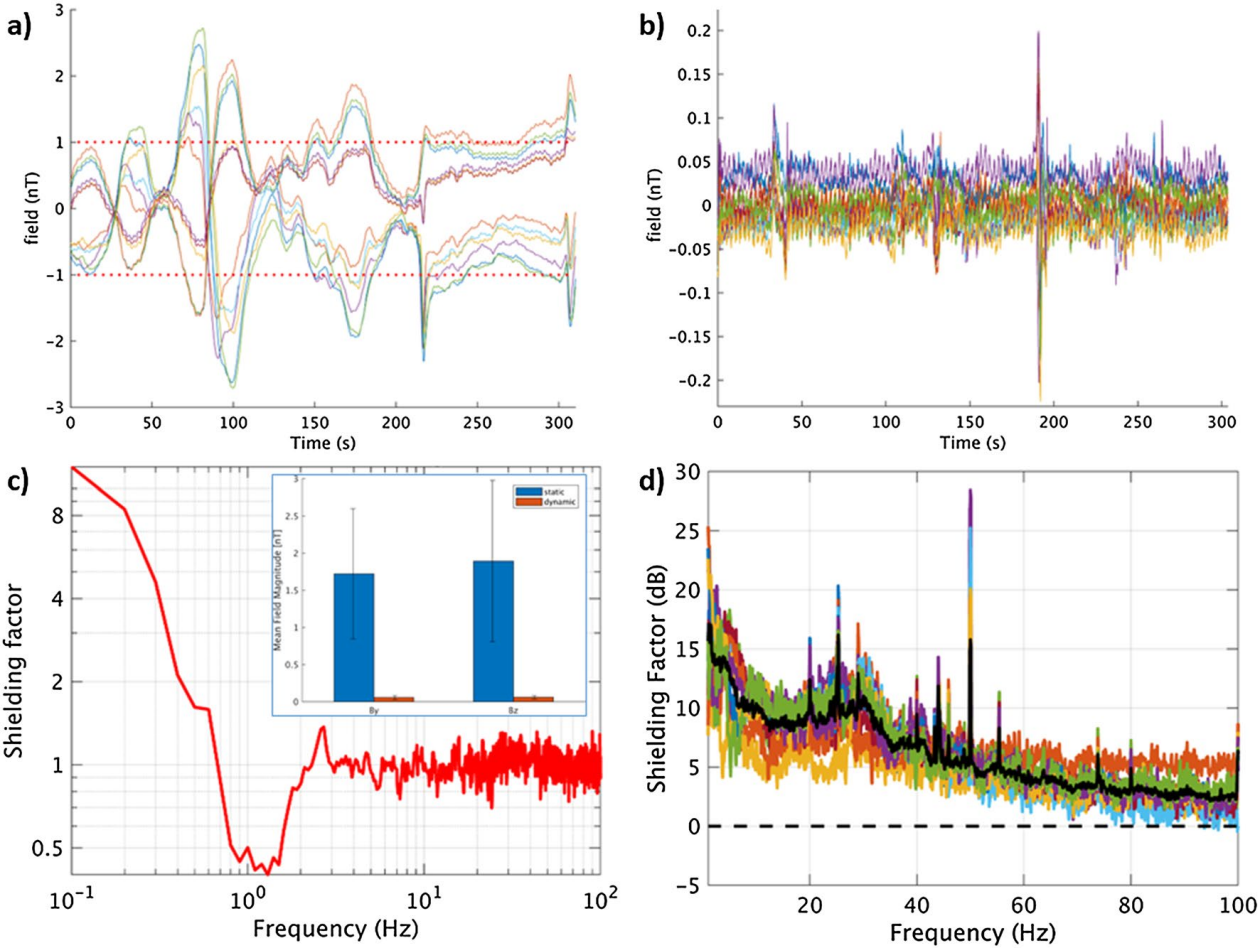
Performance of static and dynamic nulling, and homogenous field correction. Before the patient recordings, the dynamic nulling performance was quantified with a 5-min empty-room recording with the OPMs in the patient-helmet (here: patient #5). Panel (**a**) shows the data for all 12 channels with only compensation of the static remnant magnetic field (using internal and external coils). Note that the remnant fields did not remain below 1 nT throughout the recording due to fluctuations in the environmental magnetic fields. However, when dynamic nulling was applied (**b**), the change in field could be kept below 0.3 nT. The shielding factor (panel **c**; computed as the power spectral density for the dynamic nulling divided by the power spectral density for the static nulling; see also Fig. S3) was above 1 for frequencies below 0.7 Hz, with a maximum of 12 for 0.1 Hz, and approximately 1 above 2.5 Hz. In between 0.7 and 2.5 Hz the shielding factor was smaller than 1, which is due to noise that is introduced by the choice of the PID-controller’s gains. The inset shows the field magnitude (L2-norm) of the field for the 12 channels (By- and Bz-direction separately) averaged over time (with error-bars showing the standard deviation) with static (blue) and dynamic (red) nulling applied, showing that dynamic nulling decreased the field magnitude with a factor 30. The field magnitude averaged over the empty-room recordings for the 7 patients was 0.09 and 0.11 nT for By and Bz, respectively (not shown), with the maximum absolute field in a channel never exceeding 0.7 nT. Panel (**d**) shows how Homogenous Field Correction further removes noise from the recorded data (recording 1 from patient #5). The black line denotes the HFC shielding factor (in dB) averaged over all channels (coloured lines).

The output of the fluxgates was digitised using a 24-bit NI-9207 ADC module and visualised using the LabVIEW programme. Through manual adjustment of the coil-drivers the remnant fields could be reduced to ~ 1 nT, following which the fluxgates were removed and OPM recordings could be performed. Due to fluctuation in our inner-city environment, field levels would typically reach levels of ~ 4 nT again within a few minutes (Fig. 2).

Movement of the sensors through such remnant fields during a patient recording would send them outside their dynamic range, and even without such movements the remnant fields would degrade the signal fidelity through cross-axis projection errors^48^. We therefore used dynamic compensation^47,49^, based on the OPMs themselves, to reduce the remnant fields further and to keep them stable during an experiment. First, the response of the OPMs to a known current was determined by sequentially sending pulses (50 ms, 0.4 V) to the coils, resulting in an 8 (coils) × 12 (sensors) calibration matrix. During a recording, the inverted calibration matrix was used to set the required voltage outputs for the coil-drivers to minimise the sum-of-squares of the sensor outputs. A proportional–integral–derivative (PID) controller was used to drive the sensor outputs towards zero, using manually tuned proportional and integral gains (derivative gains were set to zero). The proportional and integral gain were set so that the controller responded quickly to typical changes in the remnant fields or motion artefacts, without excessive overshoot. As input to the PID, the averages of 20-sample segments of data for all 12 channels were used, sampled at 600 Hz and digitally filtered at 3 Hz with a fifth-order low-pass Butterworth filter (chosen so that the controller would not remove the (brain) signals of interest from the OPM-recordings). This reduced the maximum field changes experienced by the sensors to < 0.4 nT (Fig. 2) during empty-room recordings. During patient-recordings, the reduced static remnant fields and gradients ensured that head-movements were better tolerated^25,50^, and the dynamic compensation ensured that remnant fields/gradients remained small throughout the recordings.

#### 3D-printed helmets

**Figure 3:**
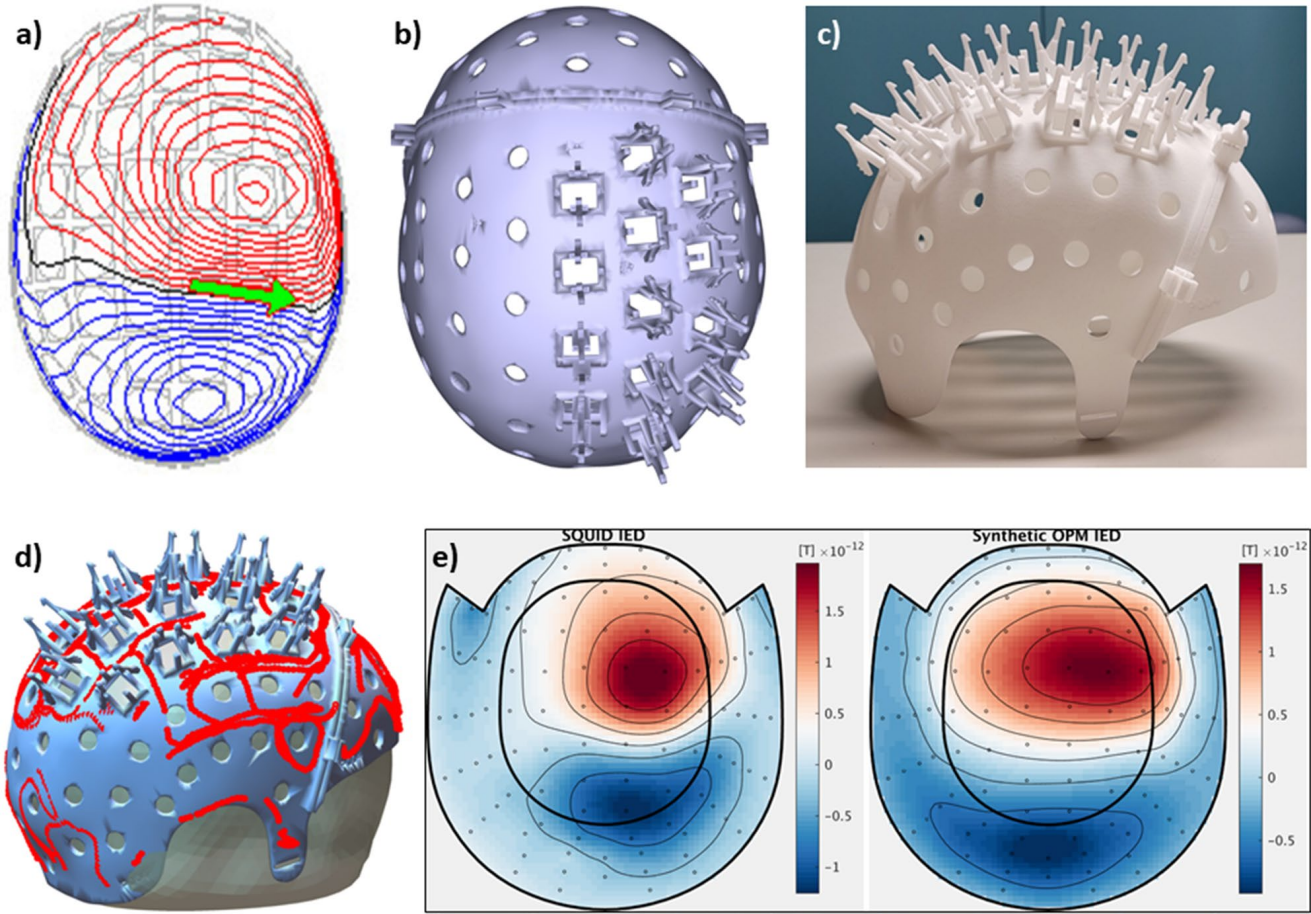
Helmet design for patient #4 and field patterns recorded with SQUIDs and OPMs. (**a**) Field pattern produced by IED (green arrow indicates the source-reconstructed equivalent current dipole) in the previously recorded clinical MEG, originating from, and in agreement with, a right central focal cortical dysplasia. (**b**) 3D-helmet model, including removable front and OPM-holders. (**c**) 3D-printed helmet. (**d**) Digitised helmet points (red dots) aligned with helmet-model, and co-registered to the anatomy (head surface from MRI). (**e**) Magnetometer field pattern for an IED (at the time point of maximum SNR) recorded with the SQUID-based system (left), as well as field pattern for an IED recorded with the OPMs, projected onto the SQUID-sensor layout (using inverse/forward projection with minimum norm^52,53^). Note the good agreement between the IED field patterns, despite the limited sampling with the OPMs, suggesting that both systems recorded similar phenomena. Also note the agreement with the previously recorded IED (panel a) (see also Fig. S5).

In order to keep the sensors firmly in place and on the scalp, individualised rigid 3D-printed helmets were constructed on the basis of the patients’ anatomical magnetic resonance images (MRIs) that were available from the clinical workup. These Digital Imaging and Communications in Medicine (DICOM) MRI files typically contained T1-weighted images recorded using a 3T scanner, with 1 mm resolution or higher. The DICOM files were uploaded in Mimics Medical 23.0 software (Materialise NV, Leuven, Belgium) and converted into 3D models using the thresholding tool: voxels with grey values within a user-specified range were included as scalp-tissue and transformed into 3D surface models using an adapted marching cube algorithm that takes partial volume effects into account. The generated model was exported in the Standard Tessellation Language (STL) file format. In Siemens NX (version 1953; Siemens AG, Munich, Germany) software a standard helmet layout (Fig. S2) was subsequently projected onto this scalp surface (for the children a 2 mm offset was added in order to account for growth during the period (2–4 years) between MRI- and OPM-scan) in order to create a patient-specific helmet-model (Fig. S2). The helmet-model contained a removable cap at the front so that the helmet could slide easily over the head, and openings for a chin strap to enable firm fixation of the helmet on the head (Fig. 3, Fig. S2). Individual OPM-holders, which included flexible side-legs to ease removal of the OPMs (modified from http://quspin.com/meg-cap-1-0) and a small ridge (2 mm) to prevent the OPMs from passing through, were manually added to the helmet-model such that the OPMs would sample the dipolar field patterns that were produced by IEDs that were identified in the previously recorded clinical MEG (Table S1). For the adult patients, the seizure onset zone, as identified in the stereo-EEG recordings, was also used to guide placement of the OPM-holders. The position of the vapour cell within the OPM casing was accounted for, which included the vapour cell’s 6.5 mm offset and the convention that OPMs were placed within the holder such that one sensitive axis was perpendicular to the head, and the other parallel to the head in the nasion-inion direction. Three reference points were added to the model to enable co-registration of the helmet with the patient’s head. The helmet-models were subsequently 3D-printed using biocompatible sintered PA12 (polyamide) nylon (Oceanz, Ede, The Netherlands). Before the patient recordings, the crosstalk between the OPMs was determined for each helmet design by sequentially activating the on-board coils and recording the responses of the remaining OPMs (see Ref.^25^ for details). The maximum crosstalk for the different helmets was on average 1.4% (range: 0.90–1.91%), and was therefore not considered during further analyses^23,25^.

#### MEG-MRI co-registration

Knowledge of the positions and orientations of the OPMs with respect to each other and the anatomy of the brain is required for source-reconstruction and array-based post-processing. The positions and orientations of the OPMs with respect to the helmet are known from the helmet modelling (assuming no errors in 3D-printing). The position of the helmet with respect to the head was determined by digitising the reference points on the 3D-printed helmet, as well as the nasion and pre-auriculars, the nose, outline of the helmet, and the forehead (when not fully covered by the helmet), using a 3D digitizer (Fastrak; Polhemus, Colchester, VT, USA). A rigid transformation of the three reference points then aligns the helmet (and OPMs) with the digitised head (Fig. 3). The digitised head was co-registered to the patient’s anatomical MRI through surface matching (using the Iterative Closest Point (ICP) algorithm^51^ in SPM (version 12)) of the head surface as extracted from the anatomical MRI with the digitised nose and forehead, or though matching of the nasion and pre-auricular points when surface matching was not possible. Combining the two transforms provides co-registration of the OPMs with the brain anatomy (Fig. 3).

#### OPM recordings

Recordings were performed in the morning in seated position, with the OPM sensors in the centre of the virtual nulling-volume (Fig. 1). Patients were instructed to sit still, with eyes closed, and were allowed to fall asleep during the recording. The calibration matrix for dynamic field compensation was determined, following which a, as per standard clinical practice^54^, 15-min OPM recording was started, as well as recording of the video-signal from the patient-monitoring system. The performance of the dynamic field compensation was monitored online, and if the noise compensation became unstable (due to large movements or a new head position that was incompatible with the calibration matrix), meaning that the feedback signal would diverge, a new recording was started. For patients with known bilateral IEDs, separate recordings were performed with OPMs over the left or right hemisphere (Table S1). For patient 7, three sensors were placed over each hemisphere. Because of the known semiology and to avoid the possibility that the patient would hurt himself or damage the OPM cabling, movement was restricted by a belt around the chest and bandages around the wrists. Recordings for this patient lasted until a seizure was recorded.

#### OPM pre-processing

Despite the dynamic field compensation, the OPM-recordings still contained interference from external (and internal) sources, as well as due to movement of the sensors through the remnant fields when the patients moved their heads. Movement-artefacts could not be regressed from the recordings^55,56^ since movements were not tracked. Sophisticated spatial filtering techniques for noise removal, such as Signal Space Separation^57^, could also not be applied to the 12 channel OPM-recordings, as the magnetic fields are undersampled and, more fundamentally, because the internal space basis set is invalid as a model of brain signals for on scalp sampling^43,58^. Tierney and colleagues have shown that as a first approximation the magnetic interference can be modelled as a spatially homogenous field, which can subsequently be regressed from the data^42,55,59^. Despite the low number of OPMs, noise was effectively removed, typically resulting in software shielding by 15 dB at low frequencies (< 2 Hz), line noise, and for artefacts with a sharp peak in the noise spectrum (24 Hz in our environment), 5–10 dB for frequencies between 2 and 50 Hz, and 5 dB and slowly declining above 50 Hz (Fig. 2).

#### OPM source reconstruction

We applied beamforming^14,60^ because of its ability to remove interference^61^ (Fig. S7) and to align with our clinical work-flow^62^, yet realising that the beamformer’s ability to localise activity will be limited with a small number of sensors. The DAiSS toolbox in SPM (version 12) was used to reconstruct the time-series of neuronal activity (so-called virtual electrodes) for the centroids^63^ of 246 regions of the Brainnetome atlas (BNA)^64^. Broadband (0.5–48 Hz) beamformer weights were constructed, for which the data covariance matrix was filtered using a discrete-cosine-transform after applying a Hanning taper, 5% Tikhonov regularisation was used when inverting the data covariance matrix. The lead fields were based on an equivalent current dipole source model with optimum orientation^65^, and a single shell head model^66^ based on the inner skull-surface of the co-registered MRI, and the homogenous field correction was taken into account^42,67^. Sensor-level data, filtered in the 3–48 Hz band with a fifth-order Butterworth filter, were subsequently projected through the normalised beamformer weights^68^.

### SQUID setup, recordings, and analyses

#### SQUID setup

The SQUID-recordings were performed with the cryogenic whole-head system, using our standard clinical protocol for epilepsy^69^. This includes the recording of two horizontal and one vertical electrooculography channel and an electrocardiography (ECG) channel, and continuous recording of the head position relative to the MEG sensors using signals from five head-localization coils. The positions of the head-localization coils and the outline of the patient’s scalp and nose (~ 4000 points) were digitized using the 3D digitizer. These scalp/nose points were used for co-registration with the head surface as extracted from the patient’s anatomical MRI, using surface matching (using ICP^70^ with in-house developed software).

#### SQUID recordings

SQUID recordings were performed on the same day as the OPM recordings, in the afternoon in supine position, and after switching off the cold-head compensation coils and bi-planar field-nulling coils. Data were recorded with a sample frequency of 1000 Hz, with an anti-aliasing filter of 330 Hz and a high-pass filter of 0.1 Hz. Internal active shielding (IAS)^71^, using MEGIN’s in-wall feedback-coils, was used for patient #1, #2, and #5, but unavailable for the other patients due to technical problems. Four datasets of 15-min duration were recorded in a task-free eyes-closed condition, during which the patients were allowed to fall asleep.

#### SQUID pre-processing

Cross-validation Signal Space Separation (xSSS)^72^ was applied to aid visual inspection of the data. Channels that were malfunctioning, for example due to excessive noise, were identified by visual inspection of the data by A.H. (mean number of excluded channels was 8, range 6–10), and removed before applying the temporal extension of SSS to the raw data (MaxFilter, version 2.2.15; Elekta Neuromag Oy)^73^, using a subspace correlation limit of 0.9 and a sliding window of 10 s.

#### SQUID source reconstruction

Our default, atlas-based beamforming implementation was used^63,74^. Elekta’s beamformer (version 2.1.28) reconstructed the time-series of neuronal activity for the centroids of the parcels in the BNA atlas. Broadband beamformer weights were computed, for which the data were filtered using a single-pass FIR filter in MaxFilter, using a Kaiser window with an order of 10,000 and 104, and attenuation of 60 dB at 0.35 Hz and 72 Hz, for the high pass (0.5 Hz) and low pass filter (48 Hz), respectively. Singular value truncation was used when inverting the data covariance matrix to deal with the rank deficiency of the data after SSS, using a truncation limit of 1e^−6^ times the largest singular value. An equivalent current dipole with optimum orientation^65^ was used as source model, and a single sphere, based on the scalp-surface of the co-registered MRI, was used as head model. The broadband data were subsequently projected through the normalised beamformer weights^68^, after which a 3 Hz high-pass filter was applied in MaxFilter (both at the sensor- and source-level) to enable comparison with the OPM data.

### IED detection and quantification

IEDs were visually identified at sensor- and source-level and marked by an experienced EEG/MEG technician (N.S.). Subsequently, an automatic algorithm (see Supplemental Material) was used to quantify the SNR of the IEDs at sensor-level, and to identify IEDs that were missed on visual inspection. A second assessor (A.H.) removed false positives from the automatically identified IEDs, using the waveforms and field maps of the visually identified IEDs as references. For the OPM data, field maps of IEDs identified in the SQUID data were used as reference and the OPM data were projected onto the SQUID-sensor layout to ease the comparison and identification of true positive IEDs (Fig. 3). In case of disagreement between assessors about true positives marked by the first assessor then these were reviewed together until a consensus was reached. All the remaining true positives were characterised in terms of Z-score (averaged over IEDs), as a proxy for SNR. Moreover, the spike-wave index (SWI) was computed, which is defined here as the percentage of seconds that contained an IED^75^.

## Results

Results of the analysis of the sensor-level OPM- and SQUID-based data are provided in Table 1.

**Table 1:** SNR and frequency of occurrence of epileptiform activity for the OPM- and SQUID-based (gradiometers only) sensor-level data. The SNR averaged (and standard deviation) over IEDs is reported. *Based on the datasets that contained epileptiform activity.

Patient	OPM		SQUID	
	SNR	SWI	SNR	SWI
#1	4.47 (0.43)	6.42	4.57 (0.46)	10.25
#2	–	0	–	0
#3	3.85 (0.32)	9.00*	3.93 (0.35)	6.76
#4	4.05 (0.48)	11.03	4.39 (0.61)	24.50
#5	–	0	–	0
#6	6.15 (0.98)	0.53*	–	0

**Figure 4:**
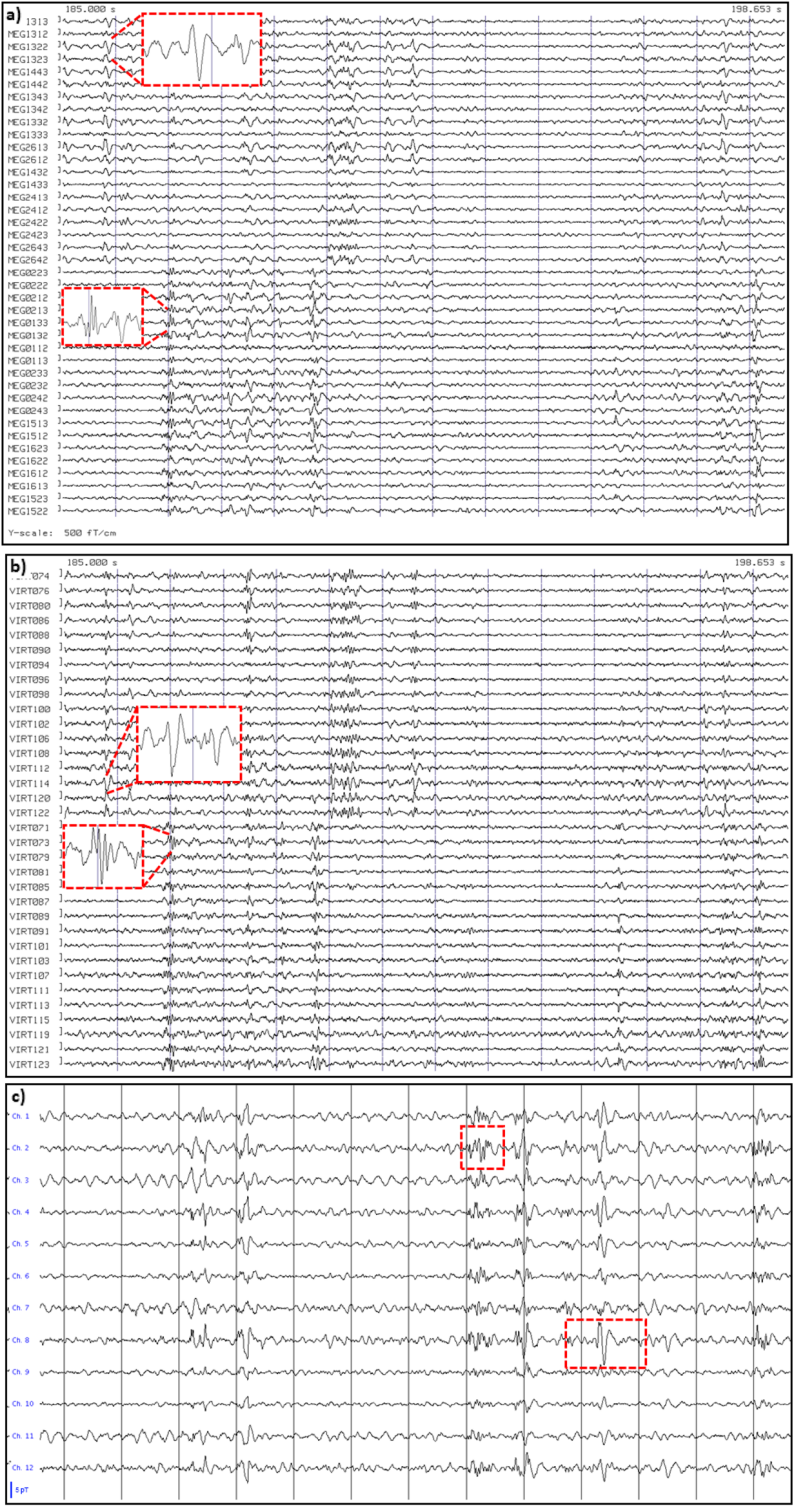

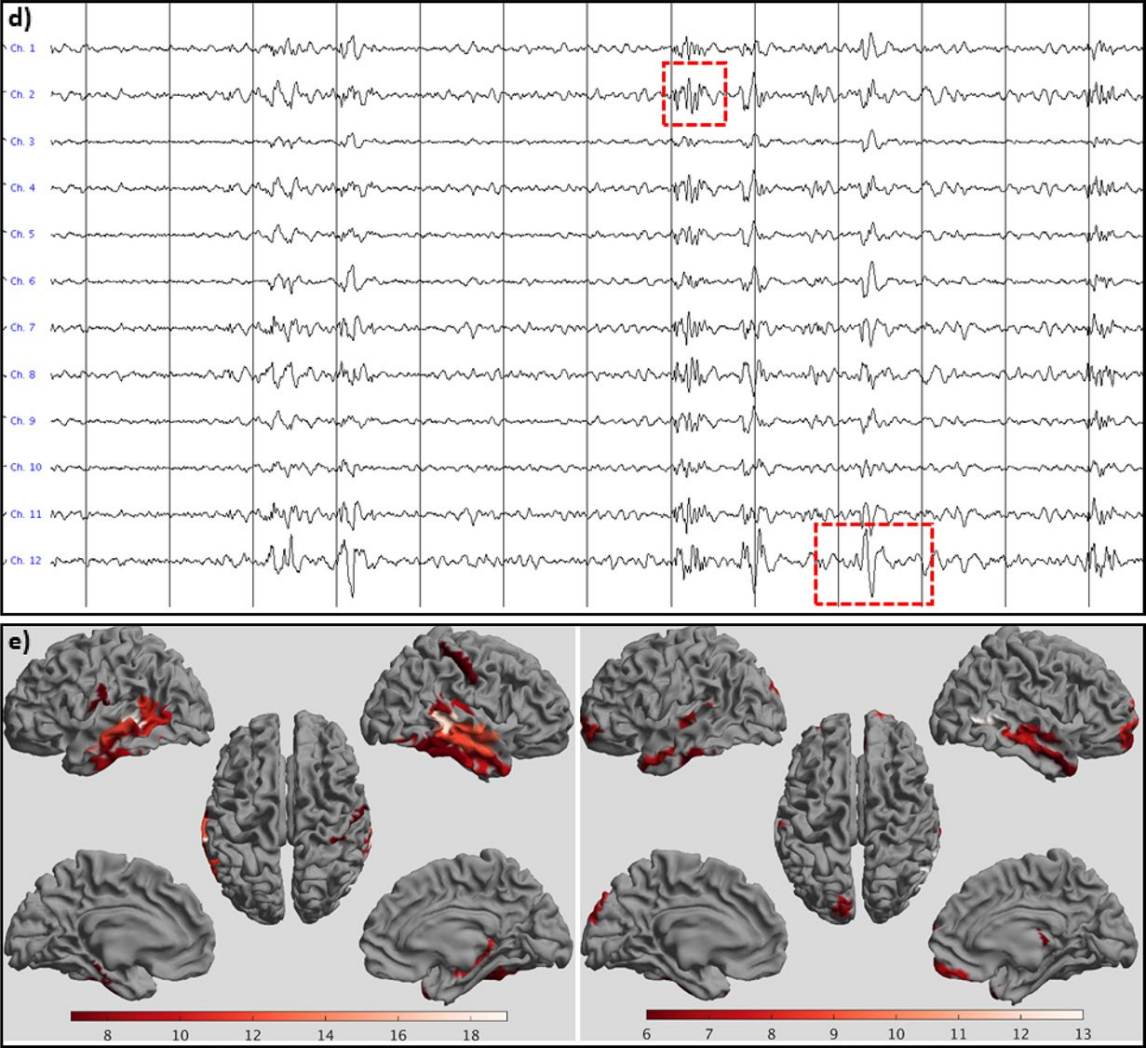
Examples of epileptiform activity for patient #1. IEDs were recorded with SQUIDs (**a**,**b**) and OPMs (**c**,**d**) at sensor-level (**a**,**c**) and source-level (**b**,**d**,**e**). (**a**) 13.653 s of data for a selection of gradiometers over the left (upper half) and right (bottom half) temporal lobes. The grey vertical lines mark 1 s of data, filtered between 3–48 Hz. Note the presence of (many) IEDs over both hemispheres, with some examples highlighted. (**b**) Virtual electrodes for a selection of the left (upper half) and right (lower half) temporal ROIs of the BNA atlas. (**c**) Comparable signals were recorded with the six OPMs, placed over the left temporal lobe in this case (recorded earlier in the day). Alternating channels show recording in the OPMs’ By and Bz direction. As for the SQUID data, some of the spike-waves and polyspikes are highlighted. (**d**) Virtual electrode data for the same data segment (selection of left temporal BNA ROIs). (**e**) Number of times a region showed the maximum SNR (over all 246 ROIs) for the events that had been identified at sensor-level (total over all datasets) for SQUID (left) and OPM data (right). Results are displayed, with an arbitrary threshold, as a color-coded map on the parcellated template brain, viewed from, in clockwise order, the left, top, right, right midline, and left midline. Note that for both systems the regions in the temporal lobes most frequently had the maximum SNR for the identified IEDs, consistent with sEEG, EEG, and earlier clinical MEG findings (see Table S1).

Patient #1 showed many short and long series of spike-wave complexes in both the OPM and SQUID data, over both the left and right temporal lobes (Fig. 4). Five recordings were performed with the OPMs over the left hemisphere (average duration 709 s; range: 306–906 s), and four with the OPMs over the right hemisphere (average duration 811 s; range: 716–901 s). SNRs were comparable, but SWI was higher for the SQUID data (10.25) than for the OPM data (6.42). This could be explained by the presence of (partly) independent left- and right-temporal IEDs in combination with the unilateral coverage during the OPM recordings: when considering only unilateral temporal channels in the SQUID data, the SWI dropped down to 6.11 for the left hemisphere, and 6.58 for the right hemisphere, and thereby became comparable to the SWI of the OPM data.

For patient #2 no IEDs were identified in either the OPM (four 15-min recordings with OPMs over the left or right hemisphere) or SQUID data.

The SQUID-recordings for patient #3 showed small IEDs, with an average SNR of 3.93 ± 0.35, over the right superior temporal/parietal lobe, often occurring in brief series (SWI = 6.76). Similar IEDs were visible on a single channel in three OPM datasets (average duration 907 s, range 900–916 s; SWI = 9.00; average SNR 3.85 ± 0.32). For the 4^th^ OPM-recording, this OPM had been moved to a new position (Fig. S4), and IEDs could not be identified in this channel anymore.

For patient #4, six OPM recordings, with an average duration of 348 s (range: 194–614 s), were performed with the OPMs over the right sensorimotor cortex. 282 IEDs were identified (SWI = 11.03; average SNR 4.05). The four SQUID-recordings revealed more IEDs (SWI = 24.50), with an average SNR of 4.39. Figure 3 shows the field configuration for IEDs recorded with both systems, illustrating that the topography is comparable across systems.

For patient #5, no IEDs were identified in either the OPM (7 recordings; on average 465 s; range 307–631 s) or SQUID data.

For patient #6, six OPM recordings were performed with the OPMs over the right central areas/superior temporal lobe. Two datasets, with a duration of 900 and 609 s, contained 3 and 5 IEDs, respectively, with an average SNR of 6.15. The other datasets (601, 540, 884, and 173 s in duration) did not contain IEDs, nor did the four 15-min SQUID-recordings. As was the case in the previously recorded clinical MEG, the SQUID data contained artefacts in right-temporal channels due to orthodontic material in the right side of the mouth, but these artefacts were largely removed by tSSS. In the OPM data, artefacts due to movement alone and those due to the orthodontic material were difficult to discern, but these were largely reduced by HFC.

**Figure 5:**
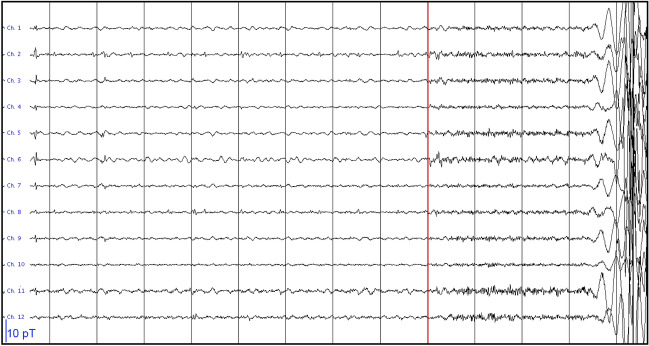
Ictal onset for patient #7. The first and last 6 channels are from the 3 OPMs over the right and left anterior temporal lobe, respectively, with alternating channels recording in the OPMs’ By and Bz direction (which is, in this case, in the anterior–posterior direction and approximately perpendicular to the scalp (inwards), respectively). The grey vertical lines mark 1 s of data, that were filtered between 3–48 Hz. Note the increase in fast activity, simultaneously over both hemispheres, after about 9 secs, marking the start of the seizure (red vertical line). This is followed by artefacts due to movement during the seizure. Although it cannot be ruled-out that the fast activity during the 3 secs before the bodily movements was due to muscle activity, we believe that this is unlikely as the clinical onset of the seizure (blinking) started ~ 2 s after the onset of the fast activity (see Supplemental Material), and no other movements were discernible during that period (compare also with video-EEG recording in Supplemental Material).

After 2.5 h, patient #7 had a seizure that was recorded with the OPM-system. The semiology was indicative of an onset in the left temporal lobe, or with a right temporal onset with rapid propagation to the left temporal lobe (see Table S1 and Supplemental Material). The seizure onset was visible in OPM sensors over both the left and right anterior temporal lobe, with no identifiable delay between the two hemispheres (Fig. 5). At source-level, seizure activity was visible in both hemispheres (Fig. S6). Despite the large amplitude movements of the patient during the seizure, approximately 30–40 cm, the OPMs stayed within their dynamic range (not shown), albeit with large movement-artefacts. Interictally, independent IEDs were observed over left and right temporal lobes, as well as simultaneously over both temporal lobes (Fig. S7), consistent with the findings from earlier sEEG recordings and seizure semiology, which pointed at independent SOZs in the left and right temporal lobe, as well as occasional rapid propagation of ictal activity between these regions. Of note, in the previously recorded SQUID-MEG IEDs were found in right fronto-temporal regions, including frontobasal and insula, but no IEDs were found in the left hemisphere. SQUID recordings were not performed during the current visit.

Based on the semiology of the recorded seizure and the striking resemblance with activity for seizures of Type 1 as previously recorded using video-EEG and sEEG (Fig. S6), the OPM data was consistent with a left temporal ictal onset and propagation, or right temporal ictal onset with rapid propagation to the left temporal lobe and further left temporal propagation.

## Discussion

The main aim of this study was to demonstrate the feasibility of OPM-based MEG in a clinical population. We showed that interictal epileptiform activity can be successfully recorded from both adults and children with challenging cases of epilepsy, and that seizure activity can also be captured. The OPM data were remarkably consistent with the data from the cryogenic system, noting that these were recorded in different sessions, with comparable SNRs and IED-yields overall. Importantly, the wearability of OPMs enabled the recording of seizure activity in a patient with hyperkinetic movements during the seizure. The observed ictal onset and semiology was in agreement with previous video- and stereo-EEG recordings. The ability to record seizures non-invasively, with high spatial resolution, is of clinical importance, as ictal activity often provides more accurate information about the epileptogenic zone than interictal activity^76^. A more accurate delineation of the epileptogenic zone may improve surgical planning and ultimately lead to improved seizure outcome in patients with refractory epilepsy.

### Performance of the two systems

The identified IEDs were consistent between the OPM- and SQUID-recordings, both in the temporal (Fig. 4) and spatial (Fig. 3) domain. Several factors could have affected the SNR and IED-yields for the two systems, making it difficult to compare their performances directly (see also Supplemental Material). The SNR of IEDs in the OPM recordings may have been affected by taking measurements along two axes, which results in a slight reduction in sensitivity (~ 30% reported by Ref.^18^, but less by Ref.^44^). Moreover, the field maps for on-scalp sensors are much more confined than for SQUID-based systems due to the reduced source-sensor distance^38^, such that IEDs’ sensor-level signatures can be easily missed, or be reduced in amplitude, when OPMs are not optimally placed. Differences in IED-yield may also be explained by state-changes, as the OPM data were recorded in the morning and the SQUID data after lunch. This, in combination with the sleep-deprivation and the difference in patient-positioning (seated versus supine), resulted in a notable increase in drowsiness in the SQUID-session, which may have affected the yield of MEG abnormalities^77^. Similarly, within a single session the IED-yield could vary over time: for patient #3, for example, the first two (out of four) SQUID-recordings contained most of the IEDs. Unfortunately, multiple technical and practical constraints, such as the size of the OPMs, their active on-board coils, and the proximity of the cold-head, did not allow for simultaneous recordings. Randomising the order of the recording sessions across patients would have avoided potential biases, but this was not feasible as setting-up the OPM-system was time-consuming. The IED-yield could also have been artificially lowered in the OPM data due to movements, with movement-related artefacts potentially obscuring IEDs during periods of movement. The patients had more freedom to move during the OPM recordings than during the (supine) SQUID recordings.

Unexpectedly, no IEDs could be found for patients #2 and #5 with either system. Patient #2 showed clear IEDs in both temporal lobes in the clinical MEG data that were recorded two and a half years prior to the current recordings, as well as in the stereo-EEG from 2 years ago. After the stereo-EEG the seizure frequency reduced, and the last seizure was a year ago, which may explain the absence of interictal activity in the current recordings. In the clinical MEG recordings of patient #5 from nearly 4 years ago there were clear spikes and polyspikes, and seizures remained, hence we do not have an explanation for the absence of IEDs in the current recordings.

### Seizure recordings

We demonstrated that seizure activity can be successfully recorded. Although the seizure onset can also be captured with cryogenic MEG^76^, OPMs have the advantage that, as long as the dynamic range of the OPMs is not exceeded during seizure-related movements, seizure propagation patterns can potentially also be reconstructed. Such patterns could be utilised to gain information about the seizure onset zone^78^, or inform modelling approaches that can aid epilepsy surgery^79^. Moreover, the wearability of the sensors opens up the possibility for long-term observations, although to capture seizures in unselected patients one often has to record continuously for many days, as is done in an Epilepsy Monitoring Unit. Currently, even with a wearable OPM-based device, the recordings are restricted to the MSR due to the limited dynamic range of the OPMs, which limits the total length of a recording session. The dynamic range can be increased though by operating the OPMs in closed-loop^80^, and by using alternative OPMs that do not rely on near-zero fields and that have a much larger dynamic range^81^. These developments could ultimately lead to long-term wearable MEG recordings in an unshielded environment, potentially removing the need for invasive stereo-EEG recordings.

### Future perspectives: increased signal quality

Ictal MEG with SQUID-based systems is feasible for selected patients (see Ref.^76^ for a review), namely those without (hyperkinetic) movements during the onset of their seizures. With OPMs the fixation to the scalp means that the movement restrictions are less severe, although movement of the OPMs through the remnant field-gradients still induces movement-artefacts (Fig. 5). However, as long as the OPMs stay within their dynamic range so that the neuronal activity is captured alongside the artefacts, there is the possibility of recovering the signals of interest. We envisage that the increased spatial sampling with whole-head OPM coverage can be leveraged to achieve this, for example using beamforming. Beamforming is a spatial filtering technique that offers the additional benefit of attenuating the contribution from ‘brain noise’ (background neuronal activity from outside the irritative or seizure onset zone), which would otherwise increase in amplitude due to the increased proximity of the sensors. Seymour and colleagues have recently shown that straightforward pre-processing, involving the regression of motion-captured movement-parameters from OPM recordings (removing artefacts < 0.5 Hz) and HFC (effectively the removal of the lower order external noise terms from SSS, removing artefacts 0–10 Hz), in combination with beamforming, allowed for movements of at least 1 m^56^. With such technical developments (see also Supplemental Material), the increased SNR of OPMs^38–40^ could be utilised to its full extent to accurately localise the SOZ non-invasively. A key attraction of OPMs compared to SQUID systems is the flexibility of sensor placement, and the ability to detect and localise the SOZ in the (mesial) temporal lobe with OPMs could be further increased by strategic placement of the sensors^82^.

### Future perspectives: helmet design

Bespoke rigid sensor arrays were created for all patients in this study (Fig. S2), based on the individual anatomical MRI, with sensor placement based on the field maps of IEDs that had been identified in the previously recorded clinical MEG, in combination with information from stereo-EEG recordings (in the adult patients). However, no further optimisation of sensor placement was performed^83–85^. Particularly for systems with a limited number of sensors, the exact location of the sensors with respect to a region of interest becomes more important^84^ (Fig. S4). As mentioned above, the field maps generated for on-scalp sensors are quite compact, and local dense non-uniform sampling yields more information than uniform sampling of a larger area^84^. In clinical practice, strong prior information about the expected generators of IEDs and ictal activity may not be available, rendering such an approach impractical. However, we envisage that multi-channel OPM-based systems with 50 + sensors and whole-head coverage^59^ will become the norm. For such systems, uniform sampling may be sufficient, as long as the sensor spacing is approximately equal to the distance of the sensors to the closest source^84^. When using triaxial sensors, 75–100 uniformly placed sensors would provide sufficient spatial sampling^43^.

### Limitations on the number of OPMs

An obvious limitation of this proof-of-principal study is the limited number of OPM sensors. By including well-characterised patients who had already undergone a successful clinical MEG that revealed IEDs that could be localised, as well as stereo-EEG (for the adult patients), we were able to increase the chances of capturing IEDs through strategic placement of the OPMs. However, despite this imbalance in the number of sensors (6 sensors/12 channels versus 102 sensors/306 channels), the OPM system’s performance was comparable to that of the SQUID-based whole-head system, in terms of SNR and IED-yield. Future studies with more OPMs are required for a comparison between systems in terms of reconstruction-accuracy of the generators of ictal and interictal epileptiform discharges.

### Limitations on differences in recording conditions and data processing

Other factors that could have affected the direct comparison between the two systems include differences in pre-processing (HFC versus SSS), head modelling (single shell versus single sphere) and beamformer implementation (DAiSS versus Elekta beamformer), as well as the differences between recording sessions mentioned above (time-of-day, seated versus supine).

### Limitations on identification of IEDs

Interictal epileptiform discharges were primarily identified on the basis of visual inspection by an experienced EEG/MEG technician. A straightforward automatic algorithm that identified brief, sharp events that clearly stood out from the baseline (i.e. had a high Z-score), was used to identify other potential IEDs that were missed on visual inspection. Due to its simplicity, our automatic detector gave many false positives when the Z-score threshold was chosen such that (most) true positives were not missed, and careful visual assessment of the identified potential IEDs was therefore still required. For example, in the sensor-level SQUID data the algorithm initially locked-on to a strong ECG artefact that was present in some channels in some of the patients. This problem was mitigated by using only a sub-selection of channels for the automatic IED identification. Similarly, some IEDs that had been visually identified were missed by the automatic detector (false negatives) because of the IED-morphology. The data from patient #1 in particular contained polyspikes/spike-wave discharges that were sometimes missed by the detector, as were some small spikes for patient #3. Use of more sophisticated algorithms for the identification of interictal abnormalities (e.g. Refs.^86,87^) would ease the objective comparison of the performance of both MEG systems, yet this was beyond the scope of the current manuscript.

A recent study showed that with the aid of artificial intelligence it is even possible to detect hippocampal epileptiform activity in the scalp EEG^88^. Simultaneous recordings of OPM- and EEG-data would enable a direct comparison between the two modalities regarding their ability to identify hippocampal IEDs. Although this is feasible^89^, it also provides considerable engineering challenges, such as placement of the sensors/electrodes and how they may affect each other. Except for patient #7, we did not perform a direct comparison between the OPM data and the EEG, stereo-EEG and/or MEG data that had been recorded previously, for several reasons: (i) the interval between these recordings was considerable (from half a year to several years), during which aging, changes in medication, or other factors could have affected the interictal activity; (ii) such a comparison, for example in terms of IED-yield, would have been biased, since OPM-placement was based on these previous recordings. It is therefore not surprising that the OPM-results, for those patients with epileptiform activity in their OPM data, were in agreement with earlier EEG, stereo-EEG and/or MEG (see Table S1).

## Conclusions

We have shown that interictal epileptiform activity can be reliably recorded with OPM-MEG, both in adults and paediatric populations. Moreover, the wearability of the sensors allowed for seizure recordings, even in the presence of significant movement. Overall, OPM data were very much comparable to those obtained with a cryogenic system, despite a potential lowering of the SNR of the IEDs due to suboptimal placement of the limited number of sensors. The relatively low cost of this technology, in combination with its reduced running and maintenance costs, means that OPM-based MEG could be used more widely than is the case with current MEG systems, and it may become an affordable alternative to scalp EEG, with the potential benefits of increased spatial accuracy, reduced sensitivity to volume conduction/field spread, and increased sensitivity to deep sources such as the hippocampus. Given its patient-friendliness, we envisage that wearable MEG will in the near future not only be used for presurgical evaluation of epilepsy patients, but also for diagnosis after a first seizure.

## Supplementary Information

Supplementary Information.

## Data Availability

Data and user-developed codes are available upon reasonable request to the corresponding author under the condition of an existing collaboration agreement.
