# Supplementary material for: Non-invasive measurements of ictal and interictal epileptiform activity using optically pumped magnetometers
Source: Sci Rep. 2023 Mar 21;13:4623. doi: 10.1038/s41598-023-31111-y (PMC10030968; doi:10.1038/s41598-023-31111-y)
Supplement: Supplementary file 1 — Supplementary Information. [file 41598_2023_31111_MOESM1_ESM.pdf]

Supplemental Material

Non-invasive measurements of ictal and interictal epileptiform activity using Optically Pumped Magnetometers

Arjan Hillebrand[a,b,c,*], Niall Holmes[d], Ndedi Sijsma[a], George C. O'Neill[e], Tim M. Tierney[e], Niels Liberton[f], Anine H. Stam[a], Nicole van Klink[g], Cornelis J. Stam[a,b,h], Richard Bowtell[d], Matthew J. Brookes[d], Gareth R. Barnes[e]

a) Amsterdam UMC location Vrije Universiteit Amsterdam, Department of Clinical Neurophysiology and Magnetoencephalography Center, Boelelaan 1117, Amsterdam, The Netherlands

b) Amsterdam Neuroscience, Brain Imaging, Amsterdam, The Netherlands

c) Amsterdam Neuroscience, Systems and Network Neurosciences, Amsterdam, The Netherlands

d) Sir Peter Mansfield Imaging Centre, School of Physics and Astronomy, University of Nottingham, University Park, Nottingham, NG7 2RD, UK

e) Wellcome Centre for Human Neuroimaging, Department of Imaging Neuroscience, UCL Queen Square Institute of Neurology, University College London, WC1N 3AR, UK

f) Amsterdam UMC location Vrije Universiteit Amsterdam, Department of Medical Technology, 3D Innovation Lab, Amsterdam, The Netherlands

g) UMC Utrecht Brain Center, University Medical Center Utrecht, Department of Neurology and Neurosurgery, Heidelberglaan 100, 3584 CX Utrecht, The Netherlands

h) Amsterdam Neuroscience, Neurodegeneration, Amsterdam, The Netherlands

* Corresponding author: a.hillebrand@amsterdamumc.nl

Semiology during video-EEG and OPM recording – Patient #7

During the video-EEG recordings, two types of seizure were recorded:

Type 1

Patient opens eyes, sits up if he was in supine position. For seizures that start when he is awake: he tries to say that he has a seizure, but does not finish sentence. Looks frightened, eyes wide-open, face contorted. Left eye a bit more closed than right eye. Generalized increased tone for seconds (e.g. visible by raising of the shoulders), followed by hyperkinetic seizure with kicking, rocking movements with body, changing between sitting and lying down. Early in the seizure there is (if right-side is visible) a dystonia of the right hand. Large amplitude circular repetitive movement of the right arm. Eventually the movements stop, following which oromandibulaire automatisms (smacking/chewing), sometimes with right-sided manual automatisms, unforced slow head deviation to the left. Sometimes some noises/audible breathing. Impaired awareness. Post-ictally he speaks little and slowly, which may be aphasia, but is difficult to differentiate from a post-ictal state. During some seizures with initial hypermotor semiology as described above, this is not followed by temporal semiology, and patient also responds earlier when he is tested.

Type 2

Oromandibulaire automatisms, with some salivation, impaired awareness. Sometimes more subtle hyperkinetic semiology (subtle movements of legs/arms/trunk), generalized increased tone, blinking. Post-ictal possibly aphasia, but also difficult to differentiate from a post-ictal state (does not name, or very slow). On EEG these seizures have a right temporal onset and right temporal propagation pattern.

During the OPM recordings, the following semiology was observed:

Starts with eyes closed. Brief (~1 sec) blinking, then moves head forwards and back. Crosses arms in front of chest, looks up, frightened, with left eye closed and right eye wide open. Hypermobility, kicking, rocking movements with body and swinging left arm. Generalized increased muscle tone, unforced slow head deviation towards left, face contorted with mouth wide-open, following which some oromandibulaire automatisms (chewing). Early in the seizure there is a dystonia of the right hand. Eventually fewer movements, and leaning backward towards the left. Then sits straight and removes bandage from left arm and wipes nose with left arm. Impaired awareness.

Remains restless for several minutes: head movements, intermitted eyes-open, fiddling with chin-strap, oromandibulaire automatisms, wipes nose with left arm.

Conclusions

The semiology observed during the OPM recording is in agreement with the semiology observed for seizures of Type 1 during video-EEG. sEEG has shown that these have a left temporal onset and propagation, or right frontal/temporal onset with rapid propagation to left temporal, with a further left temporal propagation (see also Table S1).

	Patient #1	Patient #2	Patient #3	Patient #4	Patient #5	Patient #6	Patient #7
**Age [yrs]**	34	49	48	12	12	10	23
**Age at onset epilepsy [yrs]**	24	0	11	0	7	2	13
**Seizure type**	Focal impaired awareness	Focal impaired awareness (sometimes) to bilateral tonic clonic	Focal impaired awareness (sometimes) to bilateral tonic clonic	Focal impaired awareness	Focal aware	Focal impaired awareness	Focal impaired awareness
**Medication**	Carbamazepine Levetiracetam	Carbamazepine Clobazam Levetiracetam Topiramaat	Sodium valproate Lacosamide	Clobazam Oxcarbazepine Topiramaat	Carbamazepine Clobazam	Sodium valproate Sultiam	None[#]
**Interval clinical MEG [months]**	18	31	40	17	45	38[*]	6
**Clinical MEG abnormalities**	Extensive bilateral epileptogenic network (L>R). Temporal, frontal, subcortical, insula	Focal abnormalities bilateral temporal: delta and epileptiform discharges (L>R)	Weak focal and epileptiform discharges R temporal	Focal abnormalities and epileptiform discharges R parasagittal/precentral	Focal abnormalities and epileptiform discharges R centro-parietal	Focal abnormalities and epileptiform discharges R central, during sleep changing into focal ESES. Epileptogenic network involves R postcentral gyrus, parietal inferior lobe, temporal superior gyrus and insula	Focal abnormalities temporal: delta, and theta (L>R) + epileptiform abnormalities large area R fronto-temporal, basal, insula
**MRI abnormalities**	Asymmetric gyral-sulcal pattern (R most deviant), with blurring of white-grey matter	L mesiotemporal sclerosis	Old haemorrhage L temporal, probably from small CCM	FCD R post- and partly precentral gyrus	FCD R precentral gyrus	None	Small CCM L posterior temporal

	junction, R anterior and anteromedial temporal						
**Ictal EEG onset**	Onset R temporal, then large area L temporal. Frequent spike-wave complexes over extended temporal area accompanied by (speech)arrest	Onset R temporal, with propagation to L hemisphere. UEO before UCO	Onset R parieto-central (vertex), then propagation to contralateral. UCO before UEO	Onset mid central with sharp activity, slowing down to delta activity	Onset R parietal (mainly mid parietal), then R centro-parietal with propagation to the midline and to some extent surrounding areas	Onset R central with fast propagation to frontal and parietal, and to L hemisphere	Type 1: diffuse flattening followed by muscle artefacts due to hyperkinetic movements; R frontal then L temporal propagation. UCO before UEO (apart from occasional diffuse flattening). Type 2: R temporal. UCO before UEO
**Interictal EEG abnormalities**	Epileptiform discharges over large L and R temporal area. R temporal series of 0.5 seconds 22 Hz beta activity, suspect MCD	Temporal L>R delta/theta. Epileptiform discharges (sharp slow waves) L temporal or frontal	Focal and epileptiform discharges R frontotemporal, parietal, and L frontotemporal	Epileptiform discharges mid to R central. Focal slow activity R posterior temporal	Sharp slow wave complexes centro-parietal, mainly during (falling) asleep, sporadically in awake state, also post-ictally	Frequent epileptiform discharges and continuous slow activity R central operculum. In sleep ESES	Temporal, insular and fronto-basal delta, theta (L>R). Epileptiform discharges R fronto-basal, temporal, insular. During sleep sharper, and possibly L>R
**Other imaging modalities**	Normal FDG-PET	FDG-PET: hypometabolism L temporal: hippocampus, mesial temporal and anterior temporal, extending to	Normal PET	Not performed	Not performed	FDG-PET: hypometabolism central operculum (R>L)	FDG-PET-CT: hypometabolism L fronto-basal and possibly L temporal (neocortical and hippocampus)

		neocortical temporal						
**sEEG findings**	Multifocal onset (often R temporal) in extensive bilateral epileptogenic network (R>L)<br>At least once UCO before UEO<br><br>ICES: recognizable aura symptoms on several contact points, but not consistently in one lobe or on one side (fear, feeling weird, difficult to describe). No unequivocal contact point with semiology as during spontaneous seizures	Onset in L or R hippocampus, with propagation to L hemisphere in the latter case.<br>Interictal: L>R hippocampus<br><br>ICES: L and R hippocampal focal seizure with clinically only anterograde amnesia.<br>Following stimulation of the L anterior insula electrode (contact points in the insula as well as contact points located more frontally) symptoms of dizziness occurred, which were recognized as seizure onset symptom by the patient	Extensive and bilateral epileptogenic network, with onset R parietal (MEG focus)<br><br>ICES:<br>Seizures with semiology as during spontaneous seizures, as well as subclinical seizures, following stimulation L and R hippocampus and R parietal, with propagation to R parietal. R parietal stimulation gives semiology equal to that of the start of spontaneous seizures. Many contact points with local and regional discharges, often at relatively low stimulus intensities	Not performed	Not performed	Not performed	Focal seizures independent from R and L HC (R > L). Seizures with HCL onset came with hyperkinetic movements and also occurred while awake; 1 seizure showed rapid propagation to HCR and further R temporal propagation.<br>Majority of seizures with HCR onset occurred during sleep, with more subtle semiology (although not completely stereotypical R temporal semiology, sometimes also hyperkinetic movements)<br><br>Interical epileptiform abnormalities on ~all electrodes

							ICES: R temporal lobe seizure with more temporal semiology
**Semiology**	Impaired awareness for several minutes, with postictal confusion. Periods of 1-2 days with episodes of reduced concentration and amnestic aphasia	Provocation: stress. Sometimes dizziness just before seizure. Onset arrest, non-forced head- and eye-deviation towards R. Oro-alimentary automatisms Post-ictal: agitated and long-lasting receptive- and expressive aphasia	Starts during sleep. Lightheaded and vertigo. Eye-opening, clonic movements L face, followed by L hand/arm, forced head turn to the left. Can say that he is in a bad dream. Post-ictal: Sometimes visual aura (wires of silver paper). Often micturition. Amnestic aphasia	Mainly tonic L arm symptoms, frontal hyperkinetic component, deep breathing. Indicative of pre- rather than postcentral origin	Tingling in L arm/hand and weird sensations in L arm. Tonic L arm, clonic movements of L arm, head- and eye-deviation towards L with or without spinning towards L, and sometimes clonic movements orofacial. Awareness not affected	Mainly nightly seizures 1) Hypersalivation, eyelid myoclonus, oro-alimentary automatisms, noisy breading, fluctuating awareness 2) tonic L arm sits-up, confused, sometimes motor agitation	See Supplemental Material‡
**OPM placement**	L temporal; R temporal	L temporal; R temporal	R temporal/parietal	R central	R centro-parietal	R central/superior temporal	L and R temporal
**#hours sleep before recording**	4	4	4	7	4.5	7	0

Table S1: Patient characteristics, recording preparation, and clinical information, which includes the location of IEDs in MEG and EEG, ictal EEG onset, interictal and ictal sEEG findings, PET and CT abnormalities, MRI findings, and semiology. All patients were male, and all patients slept less than usual the night before the recordings. OPM placement indicates where the OPMs were placed. L = left; R = right; CCM = Cerebral Cavernous Malformation; CT = Computed Tomography; ESES = Electrical Status Epilepticus during slow-wave Sleep; FDG-PET = Fluorodeoxyglucose-Positron Emission Tomography; HC = hippocampus; ICES = IntraCranial Electrical Stimulation; MCD = Malformation of Cortical Development; MRI = Magnetic Resonance Image; REM = rapid eye movement; UCO = unequivocal clinical onset; UEO = unequivocal electrographic onset; *contained artefacts due to orthodontic material, yet data was interpretable; #after trying many: anti-seizure medication resulted in either one or more of the following: itchiness, eczema, drowsiness, memory problems, mood disturbances, fear, and had no sustained effect on the severity or frequency of seizures; ‡on average 8 seizures/night in the weeks before the OPM recording.

Cold-head coil

The motor unit in the cryocooler generates a magnetic field of ~1 μT. At the location of the subject's head, about 1.5 meters away from the cold-head, this corresponds to a remnant field of ~300 nT. From this distance, the motor unit can be approximated as a magnetic dipole. A compensation coil was built tightly around the motor unit to generate a magnetic dipole at the same location as the motor unit, and with opposing orientation, in order to generate a compensating field pattern that matches the field pattern of the motor unit at the measurement volume. Specifically, the coil consisted of two segments, one wrapped around the motor unit and one mounted on the other side of the cold-head, that were connected in series so that the current was flowing in the same direction in both coils. The surface area of the coil was small and, therefore, a large current was needed in order to generate an opposing field of about 300 nT in the measurement volume.

Automatic identification of interictal epileptiform discharges

Interictal epileptiform discharges (IEDs) were automatically identified using a matlab-implementation of the BrainSpike algorithm in BrainWave (version 0.9.162.4; developed by C.J.S.; available from https://home.kpn.nl/stam7883/). The algorithm detects spikes in sliding 1-sec windows, using steps of half a second. The windowed-data in a channel is converted to Z-scores, based on the mean and standard deviation for that window. If an event with a maximum Z-score exceeds a threshold within the middle 500 msec of the window (a threshold of 4, 3.5, 3.5, and 4.5 was used for patient #1, #3, #4, and #6, respectively), and if the event has a minimum and maximum duration (defined as the time between zero-crossings) of 20 and 200 msec, respectively, then it is marked as a potential IED. The algorithm was re-run on negated data in order to also identify 'negative' IEDs, and the IED with the maximum Z-score within a window, and across channels, was kept. Thus, a single Z-value was obtained for each IED, which was used as a proxy for the IED's SNR.

In order to reduce the number of false positives, the algorithm was run on only a selection of channels. The channel-selection was based on the field maps of the IEDs that had already been identified on the basis of a visual analysis of the data.

Comparison between systems

The noise level of OPMs (7-13 fT/√Hz in our case) is higher than for SQUIDs (~3 fT/√Hz), yet this is compensated for by the increase in signal amplitude due to the on-scalp placement of OPMs (reducing the source-sensor distance by ~2 cm compared to fixed-helmet SQUID-based systems). Simulations[1,2] and experimental data[3,4] have shown that the SNR of OPM-based measurements is indeed higher than for SQUID-based recordings. The largest gains are found for cortical sources, as the relative reduction in source-sensor distance is larger there than for deeper brain structures.

To demonstrate the advantages of a reduced source-sensor distance we quantified and compared the SNR and IED-yield for OPM data with data from the SQUID-based system.

For each patient, the average Z-scores of all identified true positive IEDs were compared between SQUID and OPM recordings using the Mann-Whitney U test (ranksum.m in Matlab (R2018b)) with an alpha of .05. Hence, we used the assumption that the observations between the separate recordings were independent (since the recordings were performed at different times, and the IEDs themselves could therefore have changed over time). This is a conservative approach though, and one could increase statistical power by taking into account that the within-patient observations were not completely independent, for example through mixed-effects modelling[5]. Effect size was determined using Cliff's delta, and categorised as negligible ($|d| < 0.147$), small ($0.147 \leq |d| < 0.33$), medium ($0.33 \leq |d| < 0.474$), or large ($|d| \geq 0.474$).

For patient #1 the SWI was comparable between systems, after taking the unilateral OPM placement into account. The difference between the SNR of the IEDs in the OPM data and SQUID data was negligible (4.47 versus 4.57; $U = -3.81$, $d = -0.14$, $p = 1e-4$). The OPM-recordings for patient #3 revealed more IEDs (SWI = 9.00) than the SQUID-recordings (SWI = 6.76), but the difference in SNRs was negligible (3.85 versus 3.93; $U = -2.86$, $d = -0.14$, $p = .0043$). For patient #4 the SQUID-recordings revealed more IEDs (SWI = 24.50), with significantly higher SNR and medium effect size (4.39 versus 4.05; $U = 9.53$, $d = -0.36$, $p = 2e-21$). In patient #6 the OPMs detected IEDs that were not observable in the SQUID data.

Thus, for one patient the OPMs identified IEDs that were missed by the SQUID-based system, and for one patient the IED-yield was higher for the OPMs. Somewhat

surprisingly, for two patients the IED-yield was higher for the SQUIDs (for one of these patients the IED-yield was comparable when the unilateral coverage of the OPMs was taken into account), and for three patients the SNRs of IEDs were (slightly) lower in the OPM data than in the SQUID data, although with negligible effect sizes for two of these patients.

These differences in performance should be interpreted with caution though, as several factors could have affected the SNRs and IED-yields for the two systems: The recordings with the two systems were performed on the same day, but in different sessions, so state-changes could have affected the IED-yields. Moreover, the freedom to move during the OPM-recordings could have introduced movement-related artefacts that may have obscured IEDs. On-scalp OPM arrays have more focal fields-of-view[1], implying that a more exact placement with respect to the underlying sources is required in order to capture the field extrema. Using a small number of OPMs may therefore have resulted in a reduced SNR, and possibly a lower IED-yield (especially for IEDs with low SNR). Interestingly, the more focal fields-of-view also means that with equal SNR, OPMs still offer higher spatial resolution than SQUIDs. SNR may also have been affected by the use of two-axial recordings[6,7]. Besides these general considerations, other factors may have also affected the SNR and/or IED-yield:

For patient #1, clinical MEG and sEEG recordings had previously shown the involvement of an extended epileptogenic network (Table S1). Some of the IEDs in this extended network, or their field extrema, could have been missed by the small number of OPM. Moreover, the origin of the IEDs was in mesial temporal structures, for which the decreased source-sensor distance does not fully outweigh the decreased sensitivity of the OPMs compared to the SQUIDs[2]. This could be remedied in future recordings by increasing the number of OPMs, including placement of OPMs over basal areas, and/or against the roof of the mouth[8].

Somewhat surprisingly, given the superficial origin of the IEDs, the average SNR of IEDs in patient #3 was comparable between the two systems. However, the SNR of the IEDs was low for both systems, and more IEDs were observed with the OPMs than with the SQUIDs. The inclusion of more, low-amplitude, IEDs reduced the average SNR for the IEDs in the OPM data. This would lead to the conclusion that the OPMs actually outperformed the SQUIDs, by their ability to detect such weak IEDs. However, sensor placement also had a big effect on the detectability of IEDs in this patient, as displacement of one OPM by 2.8 cm (Figure S4) made the IEDs

undetectable. Another explanation for the findings could therefore be that the SNR was, also in this patient, reduced by sub-optimal placement of the OPMs.

This is also the most likely explanation for the observed lower SNR for OPMs compared to SQUIDs for patient #4, who also had IEDs with a superficial origin (Table S1, Figure 3). It should be noted that even though the SNR differed significantly for this patient, due to the large number of IEDs, the actual difference in SNR was small (i.e. within the standard deviation of the SNR for the individual systems), with a medium effect size.

Future Perspectives

Increasing signal quality

Although one of the main advantages of OPMs is their wearability, a consequence of this wearability is that signal quality is largely affected by movement of the OPMs through the remnant field-gradients. However, with appropriate preprocessing, using motion-regression, HFC, and beamforming, movements of at least 1 meter can be tolerated[9]. It may be possible to improve on this by generating a model of the remnant fields inside the MSR, and using this model to predict and remove movement-related artefacts[10]. Moreover, reducing the field gradients in custom-designed MSRs with degaussing coils[11], or by using field-nulling 'matrix-coils' that allow for accurate field control in a large volume[12], would reduce the amplitude of movement-related artefacts to begin with. Finally, closed-loop designs can be used to further reduce movement-related artefacts, either at the OPM-level using a feedback signal to drive the on-board coils (requiring tri-axial sensitivity[7]), or through the field-nulling coils by 'real-time' updating of the coil calibration matrix, which would ensure optimal field cancellation during movements.

Helmet design

Bespoke rigid sensor arrays were created for all patients (Figure S2). It is still an open question what the optimal helmet design would be for larger clinical studies. Bespoke 3D-printed helmets are optimal in the sense that the sensors are fixed at known locations and with known orientations, yet their construction is time-consuming and costly. Flexible caps on the other hand can be re-used, but recordings may be more noisy due to sensor movements/rotations, and the exact sensor locations and orientations need to be determined. A compromise may be the use of a limited number of different-sized rigid helmets, with holders in which OPMs can be pressed onto the scalp[13]. The depth of the OPMs within their holder could be measured manually, or sensor positions and orientations could be determined automatically, using either an optical approach[14] or utilising the field-nulling coils[15]. The latter approach has the advantage that any changes in the orientations of the OPM's sensitive axes due to crosstalk from neighbouring OPMs are already taken into account. These automatic approaches could also be used in combination with a design consisting of a bespoke rigid base to which a generic flexible cap with OPM-holders can be attached (https://quspin.com/experimental-meg-cap/).

Another important consideration is that with increased coverage, heat-dissipation may become an important issue, especially for long-term recordings, for the Rubidium-based sensors used in this study, although less-so for Helium-based OPMs[16] or solid-state sensors[17] that can operate at room temperatures.

Supplemental Figures

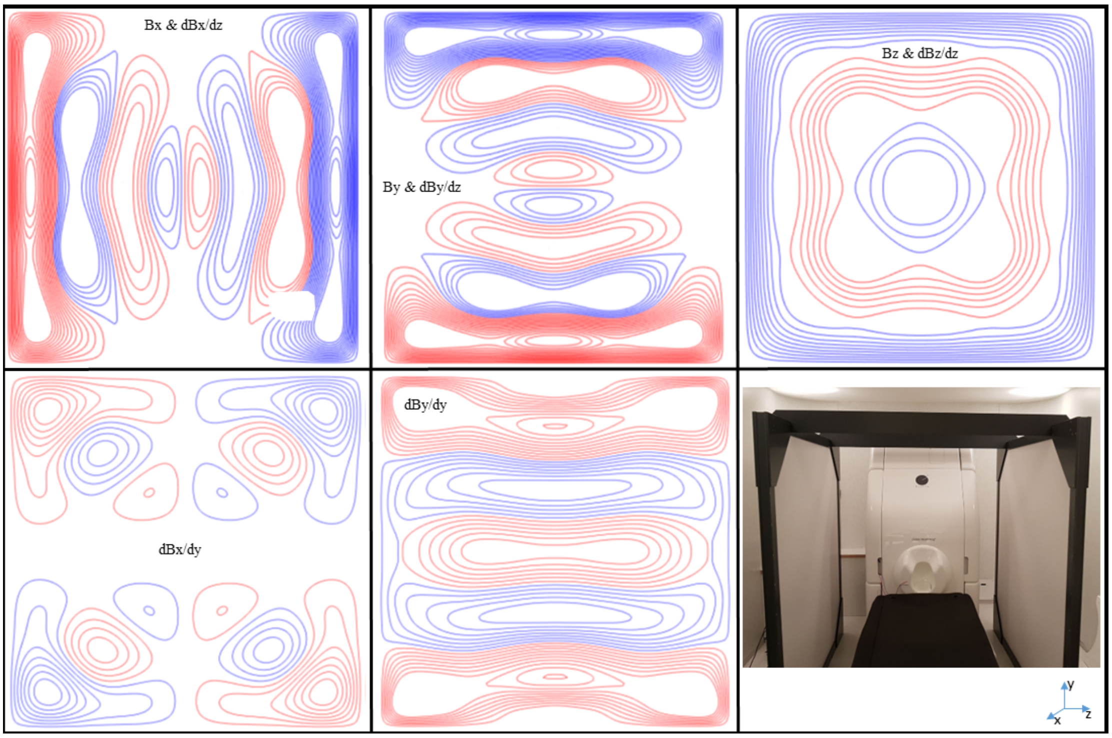

Figure S1: Bi-planar nulling coils. The natural symmetry shared by pairs of coils (Bx & dBx/dz, By & dBy/dz, and Bz & dBz/dz) was used in order to reduce the number of coils needed to minimise the field in all 3 directions, as well as the (linear) field gradients in the z-direction by a co-optimised coil design process [18]. Red and blue coloured wirepaths denote regions of opposing current flow. For the Bx & dBx/dz, By & dBy/dz coils driving the two planes with equal and opposite input currents generates a uniform magnetic field, driving the two planes with equal and same-signed currents generates a linear magnetic field gradient. For the Bz & dBz/dz coil, driving the two planes with equal and same-signed input currents generates a uniform magnetic field, driving the two planes with equal and opposite currents generates a linear magnetic field gradient. Two coils were added to allow nulling of the dBy/dy and dBx/dy gradients, in this case there is no shared symmetry which can be exploited so the windings of each plane were connected in anti-series. Grooves for each coil wirepath were routed into Forex PVC (polyvinylchloride) planes of 1.8 x 1.8 m^2. Enamelled copper wire of diameter 0.56mm was pressed into the grooves and fixed in place with tape at strategic positions. A front panel was added to prevent galvanic contact between the patients and the coils, and the stacked planes were placed inside a PVC frame (see lower-right sub-figure). The balanced coil pairs were connected individually, and the dBy/dy and dBx/dy coils in anti-series, to low-noise, 4 V, coil drivers (QuSpin Inc). Using this setup, the coils can generate uniform fields or field gradients within a 40 x 40 x 40 cm^3 volume between the centre of the panels. Bi-planar coil systems similar to those described here are commercially available (e.g. from Cerca Magnetics Limited, Nottingham, UK).

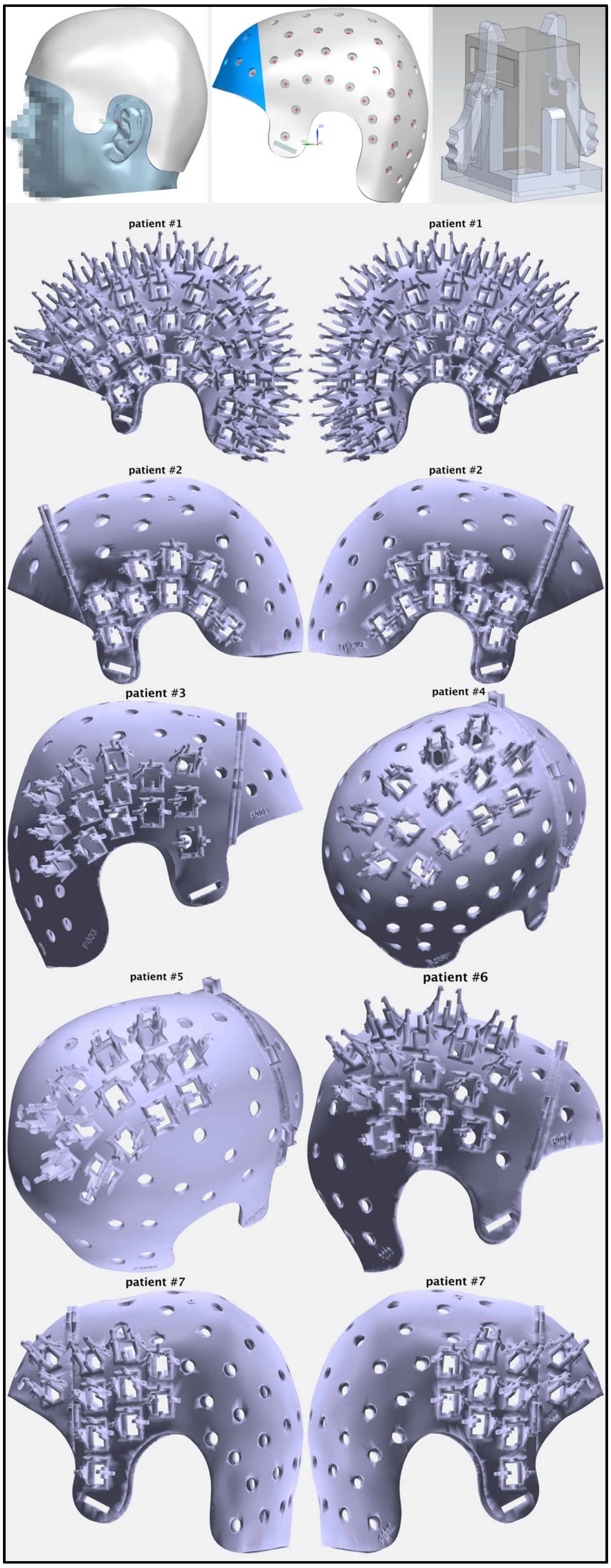

Figure S2: Helmet design, and helmet-models for all 7 patients. A 3D model of the scalp surface was created from the patient's anatomical MRI, from which the basic helmet-shape was extracted (top-left panel). A standard layout was projected onto this helmet (red markers; top-middle panel), and holes for heat-dissipation were created at these positions. A removable cap was created and holes for a chin-strap were added, as well as individual sensor-holders (top-right panel). The field maps of IEDs that had been identified in the previously recorded clinical MEG were used to guide the sensor placement (see Figure 3). That is, the sensors were placed so that the (expected) dipolar pattern would be captured with the sensors, including the two field extrema. Stereo-EEG recordings were used as gold-standard for confirmation of the location of the IEDs as identified in the previously recorded clinical MEG.

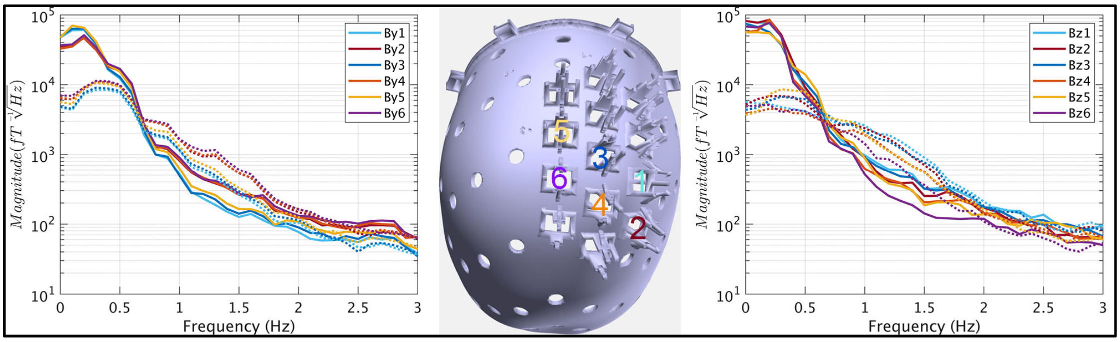

Figure S3: Power spectral densities (PSDs) for static and dynamic nulling. Power spectral densities are shown for an empty-room recording in the By (left panel) and Bz direction (right panel) (which is, in this case, in the anterior-posterior direction and approximately perpendicular to the scalp (inwards), respectively). Solid and dotted lines are for static and dynamic nulling, respectively. The middle panel shows the helmet-model, in which the OPM positions are indicated. Note that with static nulling, and below 0.5 Hz, there was a field gradient along the anterior-posterior direction for By (the PSDs for OPM 1, 3 and 5 do not overlap with those for OPM 2, 4, and 6). With dynamic nulling (dotted lines) this gradient disappeared, and the overall noise level had gone down by approximately a factor 10 (see also Figure 2).

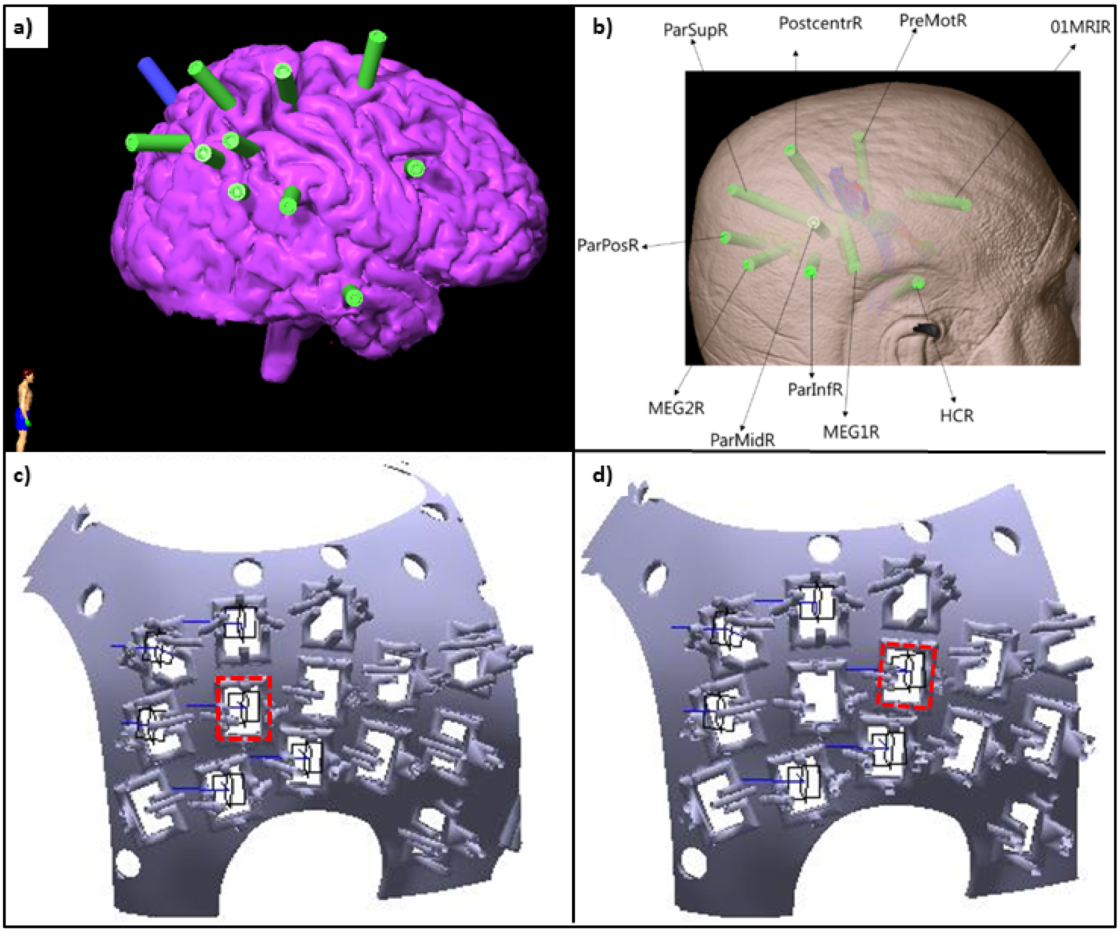

Figure S4: sEEG setup and OPM-placement for patient #3. Panels **a)** and **b)** show the depth-electrodes (green) with respect to the brain and scalp, respectively. The OPMs were placed over the right superior temporal/parietal lobe (electrode with label 'MEG1R') as this is where seizures started in the sEEG recordings, and where previous clinical MEG had localised IEDs. Using the OPM placement as in panel **c)**, the OPM that is indicated with a red rectangle recorded IEDs in the Bz channel. When this OPM was moved forward (panel **d)**), IEDs were not visible anymore. Blue arrows indicate the OPMs' sensitive axes (By and Bz), with By approximately parallel to the head and Bz approximately perpendicular to the head.

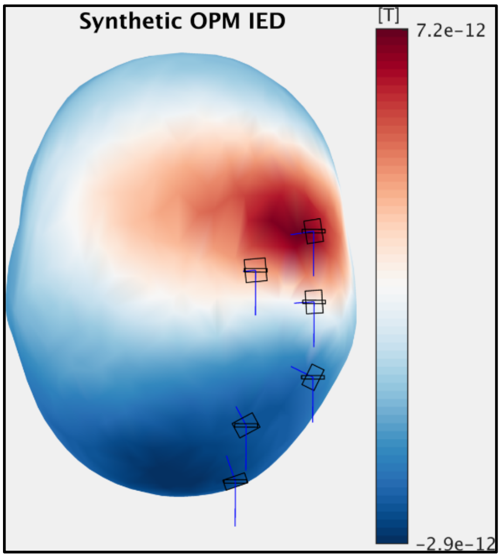

Figure S5: Field pattern recorded with OPMs for patient #4. Field pattern (in tesla) for an IED (at the time point of maximum SNR) recorded with the OPMs, projected onto the scalp (using inverse/forward projection with minimum norm). The black squares indicate the OPM positions and the blue lines the directions in which the recordings were taken. Note the agreement with the previously recorded IED (Figure 3a).

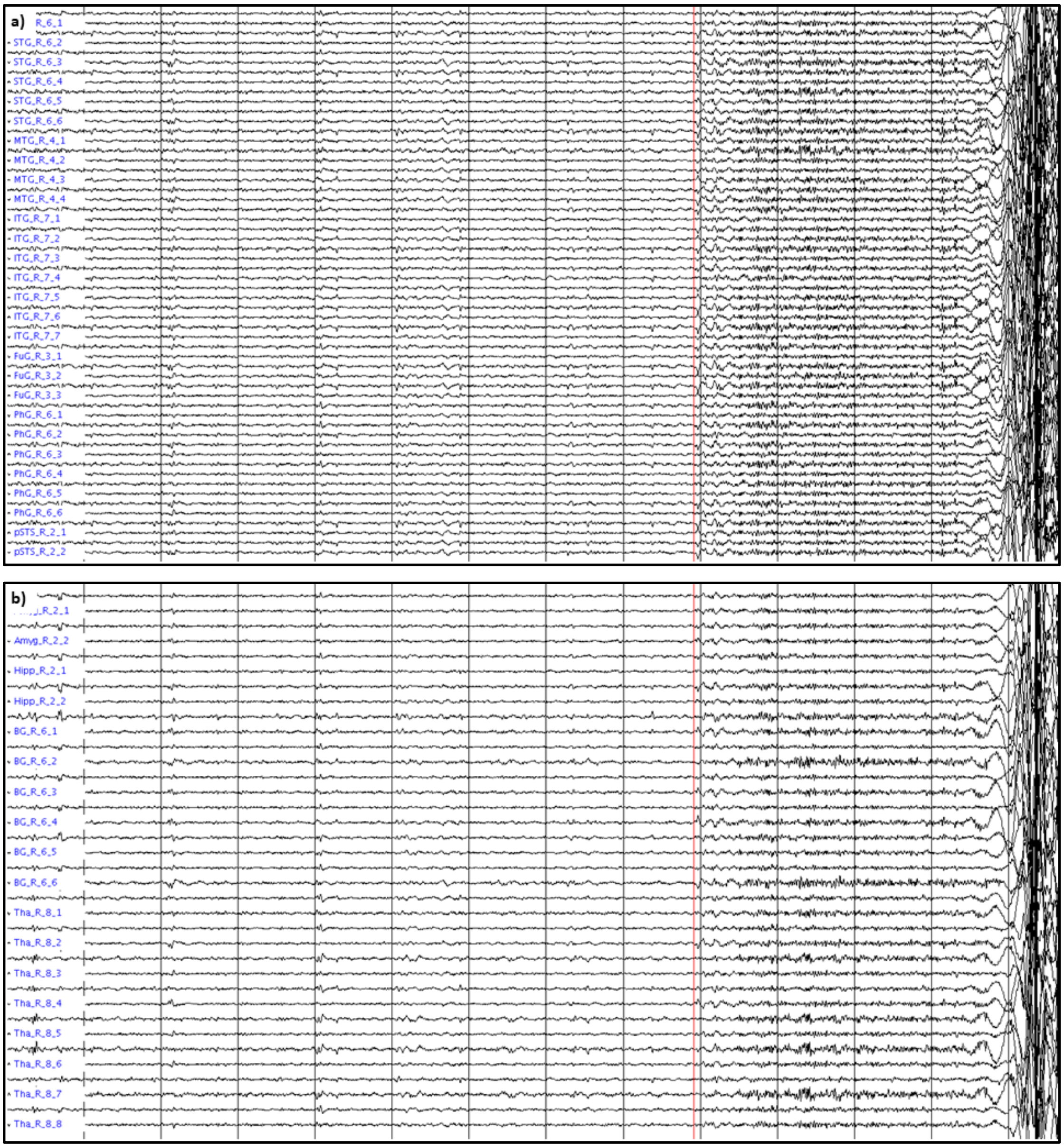
a)
R_6_1
STG_R_6_2
STG_R_6_3
STG_R_6_4
STG_R_6_5
STG_R_6_6
MTG_R_4_1
MTG_R_4_2
MTG_R_4_3
MTG_R_4_4
ITG_R_7_1
ITG_R_7_2
ITG_R_7_3
ITG_R_7_4
ITG_R_7_5
ITG_R_7_6
ITG_R_7_7
FuG_R_3_1
FuG_R_3_2
FuG_R_3_3
PhG_R_6_1
PhG_R_6_2
PhG_R_6_3
PhG_R_6_4
PhG_R_6_5
PhG_R_6_6
pSTS_R_2_1
pSTS_R_2_2
b)
R_2_1
Amyg_R_2_2
Hipp_R_2_1
Hipp_R_2_2
BG_R_6_1
BG_R_6_2
BG_R_6_3
BG_R_6_4
BG_R_6_5
BG_R_6_6
Tha_R_8_1
Tha_R_8_2
Tha_R_8_3
Tha_R_8_4
Tha_R_8_5
Tha_R_8_6
Tha_R_8_7
Tha_R_8_8

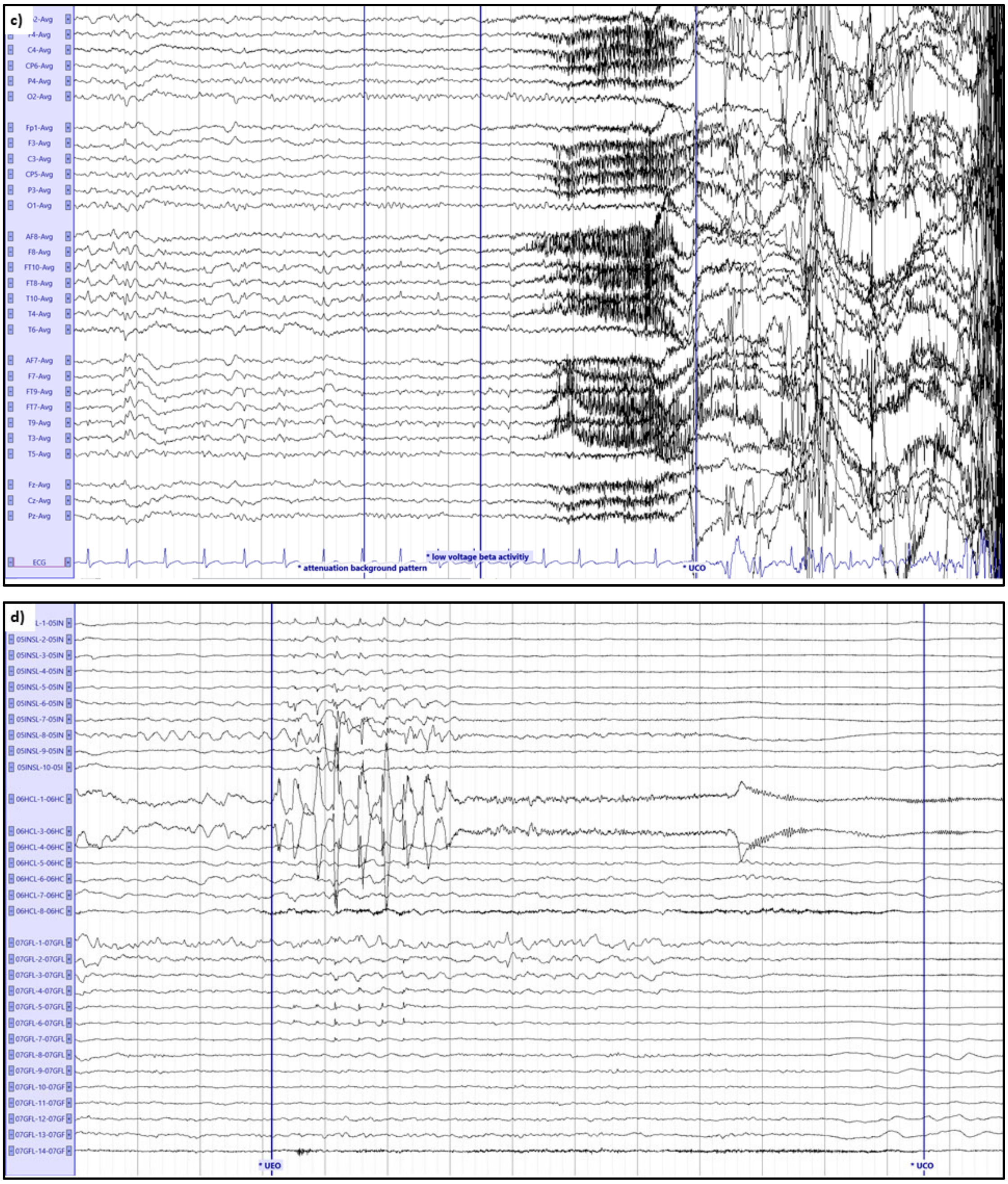

Figure S6: Ictal onset for patient #7. Source-reconstructed OPM data for temporal (**a**) and subcortical (**b**) channels in the BNA atlas (see [19] for nomenclature). Channels from left and right hemisphere are alternated. The grey vertical lines mark 1 second of data, that were filtered between 3 - 48 Hz. Note the increase in fast activity, simultaneously in both hemispheres, after about 9 secs (red vertical line), marking the start of the seizure. This is followed by artefacts due to movement during the seizure. Panel **c** shows a segment of seizure activity, Type 1, from a video-EEG recorded in 2021. Seizure from drowsy state, EEG applied according to the 10-20 system with extra electrodes from the 10-10 system (anterior frontal (AF7/8), frontotemporal (FT9/10, FT7/8), temporal (T9/10), centroparietal (CP5/6)). Average montage. The grey vertical lines mark 1 second of data, amplitude 70 µV/cm, high and low pass filter of 0.27 Hz and 15.0 Hz, respectively. The seizure starts with attenuation of the background pattern (1st blue vertical line from the left), followed with diffuse beta activity (2nd blue vertical line; mainly fronto-temporal), then beta mixed with muscle artefact (although no patient movements were visible yet), followed by large movement artefacts. Note the similarity in temporal evolution in the video-EEG and OPM recording. Panel **d** shows a segment of seizure activity, Type 1, from an sEEG recording from 2021. Only electrodes involved in the seizure onset are depicted (05INSL: entry: frontal, target: anterior insula. 06HCL: entry: temporal, target: left hippocampus. 07GFL: entry: temporal, target: left fusiform gyrus). Bipolar montage. The grey vertical lines mark 1 second of data, amplitude 700 µV/cm, high and

low pass filter of 0.27 Hz and 100 Hz, respectively. UEO: unequivocal electrographic onset. UCO: unequivocal clinical onset. Seizure onset (1st blue vertical line): 2.5 Hz spike-wave complexes on position 6HCL1-3, also visible at 5INSL1-8 and less pronounced at 7GFL1-8, followed by irregular high frequency activity and subsequent beta activity with a frequency of ~20 Hz on 06HCL1-3. Seizure propagation (not shown) in consecutive order: left posterior temporal lobe (GFL), left anterior cingulate cortex (anterior and dorsal part) and left frontobasal area.

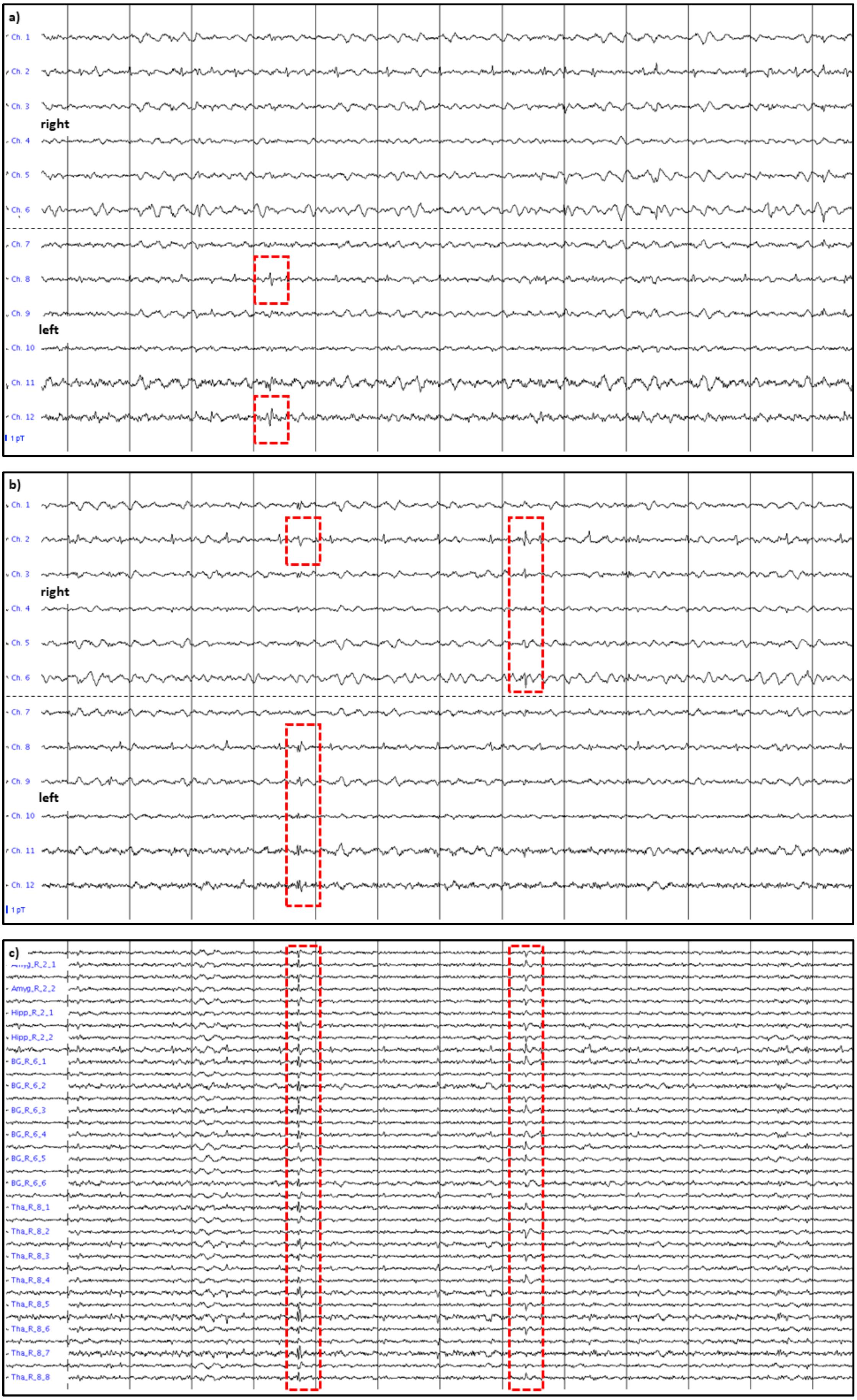
a)
Ch. 1
Ch. 2
Ch. 3
right
Ch. 4
Ch. 5
Ch. 6
Ch. 7
Ch. 8
Ch. 9
left
Ch. 10
Ch. 11
Ch. 12
1 pT
b)
Ch. 1
Ch. 2
Ch. 3
right
Ch. 4
Ch. 5
Ch. 6
Ch. 7
Ch. 8
Ch. 9
left
Ch. 10
Ch. 11
Ch. 12
1 pT
c)
Amyg_R_2_2
Hipp_R_2_1
Hipp_R_2_2
BG_R_6_1
BG_R_6_2
BG_R_6_3
BG_R_6_4
BG_R_6_5
BG_R_6_6
Tha_R_8_1
Tha_R_8_2
Tha_R_8_3
Tha_R_8_4
Tha_R_8_5
Tha_R_8_6
Tha_R_8_7
Tha_R_8_8

Figure S7: Interictal activity for patient #7. Independent IEDs were observed over the left (**a**) and right (**b**) temporal lobes, as well as simultaneously over both temporal lobes (**b**). The first 6 channels were placed over the right hemisphere and the last 6 channels over the left hemisphere; alternating channels show recording in the OPMs' By and Bz direction. Note the ECG artefact on channel 2 and 8, which was removed/reduced through beamforming (**c**). Panel **c** shows the same segment of data as in panel **b**, source-reconstructed to the subcortical channels in the BNA atlas. Channels from left and right hemisphere are alternated. The grey vertical lines mark 1 second of data, that were filtered between 3 - 48 Hz.

References

1 Iivanainen, J., Stenroos, M. & Parkkonen, L. Measuring MEG closer to the brain: Performance of on-scalp sensor arrays. *Neuroimage* **147**, 542-553, https://doi.org/10.1016/j.neuroimage.2016.12.048 (2017).

2 Boto, E. *et al.* On the Potential of a New Generation of Magnetometers for MEG: A Beamformer Simulation Study. *PLoS One* **11**, e0157655, https://doi.org/10.1371/journal.pone.0157655 (2016).

3 Boto, E. *et al.* A new generation of magnetoencephalography: Room temperature measurements using optically-pumped magnetometers. *Neuroimage* **149**, 404-414, https://doi.org/10.1016/j.neuroimage.2017.01.034 (2017).

4 Feys, O. *et al.* On-Scalp Optically Pumped Magnetometers versus Cryogenic Magnetoencephalography for Diagnostic Evaluation of Epilepsy in School-aged Children. *Radiology*, 212453, https://doi.org/10.1148/radiol.212453 (2022).

5 DeHart, W. B. & Kaplan, B. A. Applying mixed-effects modeling to single-subject designs: An introduction. *J Exp Anal Behav* **111**, 192-206, https://doi.org/10.1002/jeab.507 (2019).

6 Osborne, J., Orton, J., Alem, O. & Shah, V. Fully integrated standalone zero field optically pumped magnetometer for biomagnetism. In *Steep Dispersion Engineering and Opto-Atomic Precision Metrology XI* (eds Shahriar, S. M. & Scheuer, J.) (SPIE, 2018).

7 Rea, M. *et al.* A 90-channel triaxial magnetoencephalography system using optically pumped magnetometers. *Ann N Y Acad Sci*, https://doi.org/10.1111/nyas.14890 (2022).

8 Tierney, T. M. *et al.* Mouth magnetoencephalography: A unique perspective on the human hippocampus. *Neuroimage* **225**, 117443, https://doi.org/10.1016/j.neuroimage.2020.117443 (2021).

9 Seymour, R. A. *et al.* Using OPMs to measure neural activity in standing, mobile participants. *Neuroimage* **244**, 118604, https://doi.org/10.1016/j.neuroimage.2021.118604 (2021).

10 Mellor, S. *et al.* Magnetic Field Mapping and Correction for Moving OP-MEG. *IEEE Trans Biomed Eng* **69**, 528-536, https://doi.org/10.1109/TBME.2021.3100770 (2022).

11 Altarev, I. *et al.* Minimizing magnetic fields for precision experiments. *Journal of Applied Physics* **117**, 233903, https://doi.org/10.1063/1.4922671 (2015).

12 Holmes, N. *et al.* Naturalistic hyperscanning with wearable magnetoencephalography. *bioRxiv*, 2021.2009.2007.459124, https://doi.org/10.1101/2021.09.07.459124 (2021).

13 Zetter, R., Iivanainen, J. & Parkkonen, L. Optical Co-registration of MRI and On-scalp MEG. *Scientific Reports* **9**, 5490, https://doi.org/10.1038/s41598-019-41763-4 (2019).
14 Gu, W. *et al.* Automatic coregistration of MRI and on-scalp MEG. *J Neurosci Methods* **358**, 109181, https://doi.org/10.1016/j.jneumeth.2021.109181 (2021).
15 Iivanainen, J. *et al.* Calibration and Localization of Optically Pumped Magnetometers Using Electromagnetic Coils. *Sensors* **22**, https://doi.org/10.3390/s22083059 (2022).
16 Fourcault, W. *et al.* Helium-4 magnetometers for room-temperature biomedical imaging: toward collective operation and photon-noise limited sensitivity. *Opt Express* **29**, 14467-14475, https://doi.org/10.1364/OE.420031 (2021).
17 Koshev, N. *et al.* Evolution of MEG: A first MEG-feasible fluxgate magnetometer. *Hum Brain Mapp* **42**, 4844-4856, https://doi.org/10.1002/hbm.25582 (2021).
18 Holmes, N. *et al.* Balanced, bi-planar magnetic field and field gradient coils for field compensation in wearable magnetoencephalography. *Scientific Reports* **9**, 14196, https://doi.org/10.1038/s41598-019-50697-w (2019).
19 Fan, L. *et al.* The Human Brainnetome Atlas: A New Brain Atlas Based on Connectional Architecture. *Cereb Cortex* **26**, 3508-3526, https://doi.org/10.1093/cercor/bhw157 (2016).
